# Efficient Calculation of Electrostatic Energies for Large-Scale Nonadiabatic Molecular Dynamics in a Site Basis

**DOI:** 10.1021/acs.jctc.5c01753

**Published:** 2025-12-23

**Authors:** Samuele Giannini, Ljiljana Stojanovic, Matthew Ellis, Guido Falk von Rudorff, Jochen Blumberger

**Affiliations:** † Department of Chemistry and Industrial Chemistry, 9310 University of Pisa, Via Giuseppe Moruzzi, 56124 Pisa, Italy; ‡ Department of Physics and Astronomy and Thomas Young Centre, 4919 University College London, London WC1E 6BT, U.K.

## Abstract

Nonadiabatic molecular dynamics simulation of charge and exciton transport in molecular materials and biological systems are often carried out in a (quasi-)­diabatic or site basis. Such simulations require the calculation of the electrostatic site energy of all possible charge or excited states of the system at each molecular dynamics step, which quickly becomes computationally prohibitive when Ewald summation is used. By combining the damped shifted force real space electrostatic summation method with a suitable addition-subtraction scheme, we show that the calculation of electrostatic energy and forces for *N*_mol_ site energies can be carried out at a small and system size independent overhead compared to the calculation for a single site energy. This advance enables us to include full electrostatic interactions in nonadiabatic molecular dynamics simulations for charge and exciton transport. Applying our computational scheme to hole transport in crystalline anthracene, we find that upon inclusion of electrostatic site energy fluctuations (also sometimes termed diagonal electrostatic disorder) the inverse participation ratio measuring hole delocalization decreases from ∼5 to ∼4 concomitant with a decrease in the hole mobility by about 9% along the *b*-crystallographic direction and by 30% along the *a*-direction. Accounting for electrostatics improves the agreement with experimental time-of-flight mobilities and mobility anisotropy, but it does not alter the charge transport mechanism, transient delocalization. Our work confirms that omission of electrostatic site energy disorder is a reasonable approximation for acenes, yet electrostatics is required to obtain near-quantitative agreement with experiment, even for apolar systems.

## Graphical abstract

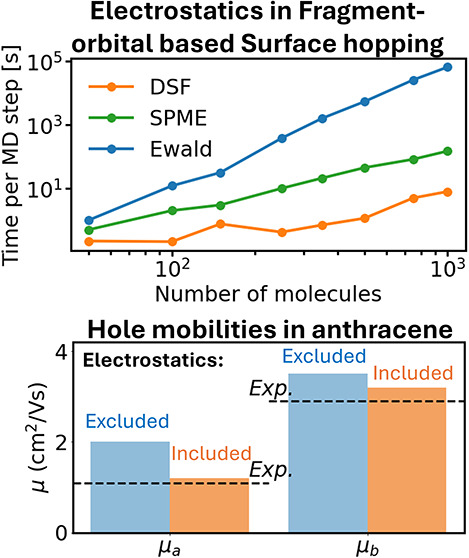

## Introduction

Electrostatic interactions are often essential for the correct description of the structure and function of condensed phase systems, in particular in polar or ionic systems including water, biomolecules and many materials. These interactions are also the most expensive energy terms to evaluate in classical molecular dynamics (MD) or Monte Carlo (MC) simulations due to their long-ranged nature. Ewald summation of the electrostatic interactions^1,2^ and its near linear scaling versions (Particle–Particle-Particle Mesh (P3M),^3^ Particle Mesh Ewald (PME)^4^ and Smooth Particle Mesh Ewald (SPME)^5^) are the defacto standard for simulations under periodic boundary conditions, which are usually applied to model (even nonperiodic) condensed phase systems. In addition, alternative methods to Ewald summation have been suggested that only require the calculation of a modified short-range interaction between atom pairs and are thus linear scaling and computationally simpler then Ewald summation as they do not require the calculation of long-range interactions.^6–8^ While these methods are not strictly equivalent to Ewald summation, some of them have been shown to give properties in good agreement with the results from Ewald summation.^8^

There are situations where the system of interest can exist in more than one charge or excited state and the electrostatic energy needs to be calculated for each of them at each MD time step or MC move, adding further to the computational cost. The calculation of free energy differences for ion charging,^9^ oxidation^10^ and proton transfer^11,12^ via thermodynamic integration are prominent examples. Here, the electrostatic energies and forces for the initial and final charge states are needed at each MD time step to propagate the classical dynamics on a mixed potential energy surface composed of the potential energy of the two charge states. Another example, on which we focus in the current work, is nonadiabatic molecular dynamics simulation of charge or exciton transport in molecular materials or in assemblies of biological cofactors. Such simulations are often implemented in a (quasi-)­diabatic or site basis requiring the calculation of electrostatic energies and forces of potentially many different charge or excited states at each MD step.

Our group has recently developed a nonadiabatic molecular dynamics simulation method for charge transport in molecular materials termed fragment orbital-based surface hopping (FOB-SH).^13–15^ This method simulates the quantum mechanical evolution of a charge carrier (excess electron or hole) in a crystalline or amorphous assembly of molecules.^16–22^ For an assembly of *N*_mol_ molecules it requires the calculation of the classical force field potential energy, also called in this context “site energy”, for *N*_mol_ charge states that only differ in the molecule that carries the charge. Calculation of the *N*_mol_ site energies would require the calculation of *N*_mol_ Ewald sums at each MD time step which is prohibitive for truly nanoscale systems (where *N*_mol_ typically >100). Hence, most previous applications of FOB-SH have been carried out on apolar molecules such as acene crystals where the electrostatic site energy could, to a good approximation, be neglected.^16–19,21,22^ However, for molecules containing polar bonds or moieties, such as the latest generation of acceptor materials in organic solar cells, this is no longer expected to be a suitable approximation.^23^ Electrostatics and polarization effects are also important at the heterojunction interface between different molecular domains,^24,25^ when charged species (like ions) are present in the system^26^ and also when simulating excited state dynamics in complex biosystems.^27–29^

The purpose of this paper is to introduce a highly efficient computational scheme for the calculation of the electrostatic site energies and forces for *N*_mol_ different charge states, which we denote “addition-subtraction scheme”. It is schematically illustrated in Figure 1. We first calculate the electrostatic site energy and forces for the system where all molecules are in the neutral charge state followed by the calculation of the correction terms that account for the fact that in each state one molecule is not neutral but charged. We show that when the addition-subtraction scheme is combined with a real-space electrostatic summation techniques such as the damped shifted force (DSF) method,^8^ the computational overhead for calculation of the electrostatic site energies and forces for the *N*_mol_ charge states relative to the calculation for a single charge state is of order *O*(*N*_mol_^0^) rather than *O*(*N*_mol_). That is, the calculation of *N*_mol_ charge states just adds a constant, system-size independent overhead to the calculation for a single charge state enabling FOB-SH nonadiabatic dynamics with full electrostatics for large systems. We show that this would also be the case for the real-space part of Ewald summation but not for the reciprocal space forces. This makes DSF our method of choice for calculation of electrostatic energies and forces in FOB-SH.

**Figure 1:**
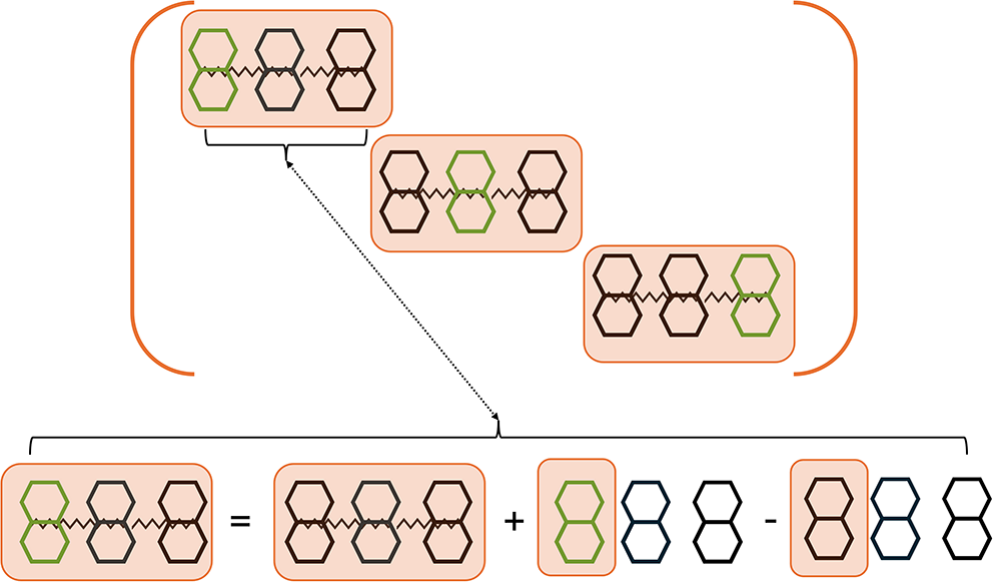
Visual depiction of the addition-subtraction scheme for calculation of electrostatic site energies and forces. The top panel shows three different charge states of an assembly of three molecules (*N*_mol_ = 3). Molecules depicted in green and black are in the charged and charge-neutral state, respectively. Wiggled lines pictorially represent nonbonded interactions. The bottom panel shows how the electrostatic energy and forces for the first of the three states is calculated using the addition-subtraction scheme. First the energy and forces are calculated for a state where all molecules are in the charge-neutral state followed by the calculation of the energy and force difference between the states where the first molecule is in the charged and charge-neutral state. When all three charge states in the upper panel are calculated this way, the computational effort is just a constant multiple of the one for the calculation of a single charge state, i.e., the overhead for calculation of *N*_mol_ charge states compared to a single charge state scales merely as *O*(*N*_mol_^0^) rather than *O*(*N*_mol_^1^).

In the following we review the electrostatic summation techniques relevant to this paper (Ewald and DSF) and explain in detail the novel addition-subtraction scheme. The energies and forces from our DSF implementation are then tested and validated against exact Ewald summation followed by a detailed analysis of the scaling behavior of the DSF addition-subtraction scheme with system size. We then use this scheme to compute important hole transfer properties in an anthracene (ANT) crystal, that is, reorganization energy from MD simulation and hole delocalization and mobility from FOB-SH nonadiabatic dynamics simulation. In this context we also present a simple charge scaling method that we use as an effective way to implicitly include electronic polarization in these simulations. We find that inclusion of electrostatics in FOB-SH simulations leads to a small decrease in the average hole delocalization and in the hole mobility that improves the agreement with experimental time-of-flight mobilities and mobility anisotropy. Importantly, we observe that the charge transport mechanism, transient delocalization,^16^ remains unchanged when electrostatics is included.

## Methods

### Ewald Summation

To set the scene, we give here the standard expression for Ewald summation of the electrostatic energy of a set of periodically replicated point charges (in atomic units) assuming conducting “tinfoil” boundary conditions^2^

1 $$E^{\mathrm{EW}}=E^{\mathrm{real}}+E^{\mathrm{rec}}+E^{\mathrm{self}}$$

2 $$E^{\mathrm{real}}=\frac{1}{2}\sum_{i=1}^{N_{\mathrm{at}}}\sum_{j=1}^{N_{\mathrm{at}}}{\sum_{n}}^{\prime }q_{i}q_{j}R^{\mathrm{EW}}\left(|R_{\mathrm{ij}}+n|\right)$$

3 $$E^{\mathrm{rec}}=\frac{2\pi }{V}\sum_{k\neq 0}\frac{1}{|k{|}^{2}}\exp\left(-\frac{|k{|}^{2}}{4\eta^{2}}\right){\left|\sum_{j=1}^{N_{\mathrm{at}}}q_{j}\exp\left(ik\cdot R_{j}\right)\right|}^{2}$$

4 $$E^{\mathrm{self}}=-\frac{\eta }{\sqrt{\pi }}\sum_{j}q_{j}^{2}$$

where in eq 2

5 $$R^{\mathrm{EW}}\left(|R_{\mathrm{ij}}+n|\right)=\frac{\mathrm{erfc}\left(\eta \left|R_{\mathrm{ij}}+n\right|\right)}{\left|R_{\mathrm{ij}}+n\right|}$$

is the short-ranged real space interaction in Ewald summation. *N*_atl_ are the number of atoms per unit cell, *q*_*i*_ and **R**_*i*_ are the point charge and position of atom *i*, **R**_*ij*_ is the position vector difference between atoms *i* and *j*, **R**_*ij*_ = **R**_*j*_ – **R**_*i*_, *i*, *j* = 1,..., *N*_at_, *V* is the volume of the unit cell, **n** are integer multiples of the lattice vectors, **k** are the reciprocal space vectors and η is a parameter determining the convergence of the real space sum, *E* ^real^, and the reciprocal space sum, *E* ^rec^. In the real space sum the ’ indicates that *i* = *j* is omitted if **n** = **0**. *E* ^self^ is a position-independent energy self-term. Notably, if the system has a total net charge of *q*, Ewald summation implicitly compensates the net charge by a homogeneous background charge of charge density −*q*/*V*. In this case a position-independent term −*q* ^2^π/(2η^2^ *V*) needs to be added to the expression eq 1. This term merely affects the absolute electrostatic energy and pressure (due to its volume dependence) but not the dynamics, structure or energy differences in NVE or NVT simulations.^30,31^

### Damped Shifted Force (DSF) Method

Several alternatives to Ewald summation have been proposed in the literature that have the advantage that they require only a pairwise real-space summation and no reciprocal space summation. Wolf et al.^6^ suggested that electrostatic interactions can be represented by modified short-ranged interactions in condensed phase systems. However, in order to converge the pairwise real space sum within a given cutoff (*R*_cut_), image charges must be used to ensure charge neutrality within the cutoff sphere. Initially, Wolf et al. ensured charge neutrality by placing image charges on the surface of the cutoff sphere. However, this led to discontinuities in the force at the cutoff radius and poor total energy conservation in MD simulations. To fix this problem Fennell et al. (building on the work of Zahn et al.)^7^ proposed the DSF technique.^8^ In this method the electrostatic energy, *E* ^DSF^ is given by

6 $$E^{\mathrm{DSF}}=\frac{1}{2}\sum_{i=1}^{N_{\mathrm{at}}}\sum_{j=1}^{N_{\mathrm{at}}}{\sum_{n}}^{\prime }q_{i}q_{j}R^{\mathrm{DSF}}\left(|R_{\mathrm{ij}}+n|\right)$$

where

$$R^{\mathrm{DSF}}\left(\left|R_{\mathrm{ij}}+n\right|\right)=\left[\frac{\mathrm{erfc}\left(\alpha \left|R_{\mathrm{ij}}+n\right|\right)}{\left|R_{\mathrm{ij}}+n\right|}-\frac{\mathrm{erfc}\left(\alpha R_{\mathrm{cut}}\right)}{R_{\mathrm{cut}}}-\left(\frac{\mathrm{erfc}\left(\alpha R_{\mathrm{cut}}\right)}{R_{\mathrm{cut}}^{2}}+\frac{2\alpha }{\sqrt{\pi }}\frac{\exp\left(-\alpha^{2}R_{\mathrm{cut}}^{2}\right)}{R_{\mathrm{cut}}}\right)\times \left(R_{\mathrm{cut}}-\left|R_{\mathrm{ij}}+n\right|\right)\right]\times \Theta \left(R_{\mathrm{cut}}-\left|R_{\mathrm{ij}}+n\right|\right)$$ 7

and Θ is the Heaviside function, truncating all interactions for atom pairs with distance larger than *R*_cut_. The first term on the right-hand side (RHS) of eq 7 is the same as the short-range real-space interaction in Ewald summation, eq 5 (where the damping parameter α replaces η). The second term is to ensure that the DSF electrostatic potential goes to zero at the cutoff radius, at |**R**_*ij*_ + **n**| = *R*_cut_. The third term, in parentheses, ensures that the DSF electrostatic force continuously tends to zero at the cutoff radius. It has been shown that the DSF method reproduces energetic and dynamic properties of liquid water and a NaCl crystal to good accuracy when compared to the results of Ewald summation. We refer to ref 8 for details.

### Multiple Charge States: Addition-Subtraction Scheme

As explained in the introduction, we would like to calculate the electrostatic interaction between a collection of atomic point charges in periodic boundary conditions for multiple charge states of the system at fixed total charge and fixed nuclear positions. As an example and without loss of generality, we consider a large periodic supercell containing *N*_mol_ identical molecules each comprised of *N*_apm_ atoms per molecule giving a total of *N*_at_ = *N*_mol_ *N*_apm_ atoms. Each molecule can exist in two charge states: a neutral state described by atomic point charges *q*_*i*_^n^ and ∑_*i*=1_^*N*_apm_^ *q*_*j*_^n^ = 0 and a charged state described by atomic point charges *q*_*i*_^c^ and ∑_*i*=1_^*N*_apm_^ *q*_*i*_^c^ = *q*, *q* typically integer, +1 or −1. Furthermore, we assume that the system can exist in *N*_mol_ different charge states, where in charge state *I*, *I* = 1,..., *N*_mol_, molecule *I* is charged and all other molecules *J* ≠ *I* are neutral. Hence, the charge states only differ in the molecule carrying charge *q* while the total charge of the supercell is equal to *q* in all *N*_mol_ charge states.

Brute-force calculation of the electrostatic energy and forces for all charge states would require *N*_mol_ Ewald sums at a computational effort that scales as *O*(*N*_mol_ *N*_at_^2^) for direct Ewald summation or *O*(*N*_mol_ *N*_at_ log­(*N*_at_)) if Ewald summation is carried out with more efficient techniques such as smooth particle-mesh Ewald (SPME). In any case, the computational cost compared to calculation for a single charge state will increase by a factor of *N*_mol_, which will become prohibitive if such calculations need to be carried out for each nuclear configuration along an MD trajectory. Here we explore a more efficient way to calculate the *N*_mol_ electrostatic energies by introducing a simple addition-subtraction scheme, Figure 1. The idea is that it may be more efficient first to calculate the electrostatic interaction energy of the state where all molecules are charge neutral and then correct for the fact that one molecule is charged. The hope is that the calculation of the correction term for the *N*_mol_ different charge states can be done at a similar effort as the calculation of the total electrostatic energy for a single charge state, thus adding a small overhead to the calculation of a single charge state. As we will see in the following, this can be achieved for DSF electrostatic energy and forces and also for the Ewald energy but not for Ewald forces.

#### Addition-Subtraction with Ewald Summation

Considering Ewald summation first, the addition-subtraction scheme gives for the real space sum eq 2

$$E_{I}^{\mathrm{real}}=\frac{1}{2}\overset{N_{\mathrm{at}}}{\underset{i=1}{\sum }}\overset{N_{\mathrm{at}}}{\underset{j=1}{\sum }}{\underset{n}{\sum }}^{\prime }q_{i}^{n}q_{j}^{n}R\left(|R_{\mathrm{ij}}+n|\right)+\frac{1}{2}\overset{N_{\mathrm{apm}}}{\underset{i\in I}{\sum }}\overset{N_{\mathrm{apm}}}{\underset{j\in I}{\sum }}{\underset{n}{\sum }}^{\prime }\left(q_{i}^{c}q_{j}^{c}-q_{i}^{n}q_{j}^{n}\right)R\left(|R_{\mathrm{ij}}+n|\right)+\frac{1}{2}\overset{N_{\mathrm{apm}}}{\underset{i\in I}{\sum }}\overset{N_{\mathrm{at}}-N_{\mathrm{apm}}}{\underset{j\notin I}{\sum }}\underset{n}{\sum }\left(q_{i}^{c}-q_{i}^{n}\right)q_{j}^{n}\left(1+\delta_{n0}\right)\times R\left(|R_{\mathrm{ij}}+n|\right)$$ 8

where *R* ≡ *R* ^EW^ given by eq 5, *I* is the index of the charged molecule, and δ_**n0**_ = 1 if **n** = **0** and zero otherwise. The first term on the RHS of eq 8 is the real space part of the electrostatic energy of the neutral system and the second and third terms on the RHS are the intramolecular and intermolecular corrections accounting for the fact that molecule *I* is charged, not neutral. The computational effort for the first term on the RHS of eq 8 scales as *O*(*N*_at_ *N*_at_^cut^) where *N*_at_^cut^ is the average number of atoms within a sphere of radius *R*_cut_^EW^ from a given atom. Here, we have assumed that the convergence parameter η is chosen such that the short-ranged interaction *R* ^EW^ can be neglected for any atom pair with distance larger than a cutoff distance *R*_cut_^EW^. Since *R* ^EW^ decays exponentially with distance, this is possible to any desired accuracy. The calculation of the second and third terms scales as *O*(*N*_apm_ *N*_at_^cut^). The first term only needs to be calculated once, whereas the second and third terms need to be calculated for each of the *N*_mol_ different charge states at *O*(*N*_mol_ *N*_apm_ *N*_at_^cut^) = *O*(*N*_at_ *N*_at_^cut^). Hence, the computational effort for *N*_mol_ real-space sums scales as *O*(*N*_at_ *N*_at_^cut^). The same scaling considerations hold true for the real-space forces

$$F_{I,k}^{\mathrm{real}}=-\frac{dE_{I}^{\mathrm{real}}}{dR_{k}}=\{\begin{array}{l} -\left[q_{k}^{n}\overset{N_{\mathrm{at}}}{\underset{i\neq k}{\sum }}\underset{n}{\sum }q_{i}^{n}\frac{\partial }{\partial R_{k}}R\left(|R_{\mathrm{ik}}+n|\right)+\overset{N_{\mathrm{apm}}}{\underset{i\in I,i\neq k}{\sum }}\underset{n}{\sum }\left(q_{k}^{c}q_{i}^{c}-q_{k}^{n}q_{i}^{n}\right)\frac{\partial }{\partial R_{k}}\times R\left(|R_{\mathrm{ik}}+n|\right)+\frac{1}{2}\overset{N_{\mathrm{at}}-N_{\mathrm{apm}}}{\underset{j\notin I}{\sum }}\underset{n}{\sum }\left(q_{k}^{c}q_{j}^{n}-q_{k}^{n}q_{j}^{n}\right)\left(1+\delta_{n0}\right)\frac{\partial }{\partial R_{k}}R\left(|R_{\mathrm{kj}}+n|\right)\right],\mathrm{for},k\in I\\ -\left[q_{k}^{n}\overset{N_{\mathrm{at}}}{\underset{i\neq k}{\sum }}\underset{n}{\sum }q_{i}^{n}\frac{\partial }{\partial R_{k}}R\left(|R_{\mathrm{ik}}+n|\right)+\frac{1}{2}\overset{N_{\mathrm{apm}}}{\underset{i\in I}{\sum }}\underset{n}{\sum }\left(q_{i}^{c}q_{k}^{n}-q_{i}^{n}q_{k}^{n}\right)\left(1+\delta_{n0}\right)\frac{\partial }{\partial R_{k}}R\left(|R_{\mathrm{ik}}+n|\right)\right],\mathrm{for},k\notin I \end{array}$$ 9

where for the derivatives $\frac{\partial }{\partial R_{k}}R\left(|R_{\mathrm{kj}}+n|\right)\equiv \frac{\partial }{\partial R_{k}}R^{\mathrm{EW}}\left(|R_{\mathrm{kj}}+n|\right)$ we get

$$\frac{\partial }{\partial R_{k}}R^{\mathrm{EW}}\left(|R_{\mathrm{kj}}+n|\right)=\left[\frac{\mathrm{erfc}\left(\eta \left|R_{\mathrm{kj}}+n\right|\right)}{|R_{\mathrm{kj}}+n{|}^{2}}+\frac{2\eta }{\sqrt{\pi }}\frac{\exp\left(-\eta^{2}|R_{\mathrm{kj}}+n{|}^{2}\right)}{\left|R_{\mathrm{kj}}+n\right|}\right]\times \frac{R_{\mathrm{kj}}+n}{\left|R_{\mathrm{kj}}+n\right|}$$ 10

with the property $\frac{\partial }{\partial R_{k}}R^{\mathrm{EW}}\left(|R_{\mathrm{kj}}+n|\right)=-\frac{\partial }{\partial R_{j}}R^{\mathrm{EW}}\left(|R_{\mathrm{kj}}+n|\right)=\frac{\partial }{\partial R_{k}}R^{\mathrm{EW}}\left(|R_{\mathrm{jk}}-n|\right)$ .

We now turn to the reciprocal space part of the Ewald sum, eq 3, which in the addition-subtraction scheme is written as follows

$$E_{I}^{\mathrm{rec}}=\frac{2\pi }{V}\underset{k\neq 0}{\sum }\frac{1}{|k{|}^{2}}\exp(-\frac{|k{|}^{2}}{4\eta^{2}})\times {\left|\overset{N_{\mathrm{at}}}{\underset{j=1}{\sum }}q_{j}^{n}\exp\left(ik\cdot R_{j}\right)+\overset{N_{\mathrm{apm}}}{\underset{i\in I}{\sum }}\left(q_{i}^{c}-q_{i}^{n}\right)\exp\left(ik\cdot R_{i}\right)\right|}^{2}$$ 11

The sum over *N*_at_ atoms on the RHS of eq 11 is only calculated once at a cost *O*(*N_k_N*_at_), where *N_k_* are the number of reciprocal space vectors included in the sum. The sum over *N*_apm_ atoms on the RHS of eq 11 needs to be calculated *N*_mol_ times which scales as *O*(*N_k_N*_apm_ *N*_mol_) = *O*(*N_k_N*_at_). Hence, the computational effort for *N*_mol_ reciprocal space sums scales as *O*(*N_k_N*_at_). Thus, the scaling is as good as for the real-space sum above. However, the same does not hold true for the reciprocal space forces which take the form

12 $$F_{I,k}^{\mathrm{rec}}=-\frac{dE_{I}^{\mathrm{rec}}}{dR_{k}}=\{\begin{array}{l} -q_{k}^{c}f_{I,k}^{\mathrm{rec}},\mathrm{for},k\in I\\ -q_{k}^{n}f_{I,k}^{\mathrm{rec}},\mathrm{for},k\notin I \end{array}$$

13 $$f_{I,k}^{\mathrm{rec}}=\frac{4\pi }{V}\sum_{k\neq 0}\frac{1}{|k{|}^{2}}\exp\left(-\frac{|k{|}^{2}}{4\eta^{2}}\right)\left[\sum_{j=1}^{N_{\mathrm{at}}}q_{j}^{n}\sin\left(k\cdot R_{\mathrm{kj}}\right)+\sum_{j\in I}^{N_{\mathrm{apm}}}\left(q_{j}^{c}-q_{j}^{n}\right)\sin\left(k\cdot R_{\mathrm{kj}}\right)\right]k$$

As for the energy above, the sum over *N*_at_ atoms on the RHS of eq 13 is only calculated once but at a cost that scales as *O*(*N_k_N*_at_^2^) as it requires the calculation of the *N*_at_ scalar products **k**·**R**_*kj*_ for each of the *N*_at_ atoms *k* and for each of the *N_k_* reciprocal space vectors **k**. The sum over *N*_apm_ atoms on the RHS of eq 13 needs to be calculated, in addition, for each of the *N*_mol_ charge states which scales as *O*(*N_k_N*_apm_ *N*_at_ *N*_mol_) = *O*(*N_k_N*_at_^2^). Hence, the computational effort for the reciprocal space forces scales unfavorably, as *O*(*N_k_N*_at_^2^), which makes the calculation of *N*_mol_ reciprocal space sums prohibitively expensive.

#### Addition-Subtraction with DSF

The interaction term in DSF is of a similar form as the short-ranged real space part of the Ewald sum, suggesting that the DSF method should exhibit a similarly good scaling behavior with respect to number of charge states as the real space Ewald sum. Using the addition-subtraction approach the DSF electrostatic energy for a given charge state is given by eq 8 with the real space interaction *R* given by *R* ^DSF^ of eq 7. *R* ^DSF^ can be calculated at the same cost as *R* ^EW^, hence the same scaling considerations apply, that is, the *N*_mol_ charge states can be calculated at *O*(*N*_at_ *N*_at_^cut^). The same holds true for the DSF forces, which in the addition-subtraction approach are given by eq 9 with derivatives $\frac{\partial }{\partial R_{k}}R\left(|R_{\mathrm{kj}}+n|\right)\equiv \frac{\partial }{\partial R_{k}}R^{\mathrm{DSF}}\left(|R_{\mathrm{kj}}+n|\right)$

$$\frac{\partial }{\partial R_{k}}R^{\mathrm{DSF}}\left(\left|R_{\mathrm{kj}}+n\right|\right)=\left[\frac{\mathrm{erfc}\left(\alpha \left|R_{\mathrm{kj}}+n\right|\right)}{|R_{\mathrm{kj}}+n{|}^{2}}+\frac{2\alpha }{\sqrt{\pi }}\frac{\exp\left(-\alpha^{2}|R_{\mathrm{kj}}+n{|}^{2}\right)}{\left|R_{\mathrm{kj}}+n\right|}-\left(\frac{\mathrm{erfc}\left(\alpha R_{\mathrm{cut}}\right)}{R_{\mathrm{cut}}^{2}}+\frac{2\alpha }{\sqrt{\pi }}\frac{\exp\left(-\alpha^{2}R_{\mathrm{cut}}^{2}\right)}{R_{\mathrm{cut}}}\right)\right]\frac{R_{\mathrm{kj}}+n}{\left|R_{\mathrm{kj}}+n\right|}\times \Theta \left(R_{\mathrm{cut}}-\left|R_{\mathrm{kj}}+n\right|\right)$$ 14

that have the property

$$\frac{\partial }{\partial R_{k}}R^{\mathrm{DSF}}\left(|R_{\mathrm{kj}}+n|\right)=-\frac{\partial }{\partial R_{j}}R^{\mathrm{DSF}}\left(|R_{\mathrm{kj}}+n|\right)=\frac{\partial }{\partial R_{k}}R^{\mathrm{DSF}}\left(|R_{\mathrm{jk}}-n|\right)$$

Thus, the DSF method allows one to calculate the electrostatic interaction for *N*_mol_ charge states with little overhead compared to calculation for a single charge state. The computational effort for the calculation of the electrostatic energy for *N*_mol_ charge states is merely a system size-independent multiple of the one for a single charge state calculation, and not a factor *N*_mol_ times more expensive than that. Thus, the computational overhead relative to the calculation of a single charge state/electrostatic sum is predicted to scale as *O*(*N*_mol_^0^) instead of *O*(*N*_mol_). In Results section we will benchmark the accuracy of DSF against Ewald summation for calculation of electrostatic energy and forces and reorganization energy, and showcase the performance of the DSF addition-subtraction in FOB-SH nonadiabatic molecular dynamics simulations, where *N*_mol_ charge states need to be calculated at each time step.

### Reorganization Energies

Reorganization energy is an important property determining, in concert with other factors, the mechanism and speed of charge and exciton transport. Simply speaking, it is the energy required to distort the nuclear geometry of a molecule from the equilibrium geometry of an initial charge transfer state to the equilibrium geometry of the final charge transfer state while staying on the same (diabatic) potential energy surface. We investigate this property in detail for two reasons. First, it provides an opportunity to assess the accuracy of DSF relative to exact Ewald summation in describing the structural and energetic response of a system upon charging or discharging of a molecule interacting with an environment. Second, recent work^32^ suggests that the external part of the reorganization energy, which is related to the electrostatic interaction of the molecule with the environment, is not entirely negligible for apolar systems, as is often assumed. The DSF addition-subtraction scheme now allows us to investigate the importance of electrostatic external reorganization energy on charge transport. In the following we present some of the formal definitions for reorganization energy that will be referred to in Results section, for a detailed treatment we refer to ref 33.

In the limit of linear response, which is usually a very good approximation for condensed phase systems, the reorganization free energy for electron transfer between two molecules interacting with an environment (solid, liquid etc.) is defined as

15 $$\lambda^{\mathrm{st}}=\langle \Delta E\rangle_{A}$$

where Δ*E* is the vertical energy gap

16 $$\Delta E\left(R\right)=E_{B}\left(R\right)-E_{A}\left(R\right)$$

*E*_A_ and *E*_B_ are the potential energies of the initial (A) and final (B) diabatic electron transfer (ET) state depending on all nuclear coordinates of the system, **R**, and ⟨...⟩_A_ denotes the thermal average on the potential energy surface *E*_A_. For simplicity, we have assumed in eq 15 that the free energy difference for ET is zero, as it is the case for ET between two equivalent molecules in the assembly. The superscript “st” refers to the fact that this reorganization energy can be obtained experimentally from the Stokes shift. Alternatively, reorganization energy may be defined by the variance (var.) of the fluctuations of the energy gap, σ_A_^2^

17 $$\lambda^{\mathrm{var}}=\frac{\sigma_{A}^{2}}{2k_{B}T}$$

where

18 $$\sigma_{A}=\langle {\left(\Delta E-\langle \Delta E\rangle_{A}\right)}^{2}\rangle_{A}$$

*k*_B_ is the Boltzmann constant and *T* the temperature. One can show that in the limit of linear response, λ^st^ = λ^var.^. Moreover, in the literature, one often encounters reorganization energies obtained from so-called 4-point (4p) calculations

19 $$\lambda^{4p}=\left[E_{A}\left(R_{B}\right)+E_{B}\left(R_{A}\right)\right]-\left[E_{B}\left(R_{B}\right)+E_{A}\left(R_{A}\right)\right]$$

where **R**_A_ and **R**_B_ are the nuclear coordinates at the minimum of the potential energy surfaces *E*_A_ and *E*_B_, respectively. They can be considered as the zero temperature limit to the Stokes reorganization energy, lim_*T*→0_λ^st^(*T*) = λ^4p^. It is also common practice to break down the reorganization energies λ^*x*^, *x* = st, 4p, in an inner-sphere or internal contribution due to intramolecular reorganization of electron donor and acceptor molecule, λ_i_^*x*^, and an outer-sphere or external contribution due to reorganization of the rest of the system, λ_o_^*x*^

20 $$\lambda^{x}=\lambda_{i}^{x}+\lambda_{o}^{x}$$

Marcus also made this distinction and derived for the outer sphere contribution an analytic expression using electrostatic continuum (c) theory^34^

21 $$\lambda_{o}^{c}={\left(\Delta q\right)}^{2}\left(\frac{1}{\epsilon_{\mathrm{op}}}-\frac{1}{\epsilon_{s}}\right)\left(\frac{1}{2r_{D}}+\frac{1}{2r_{A}}-\frac{1}{R}\right)$$

where ϵ_op_ and ϵ_s_ are the optical and static dielectric constant of the medium, respectively, *r*_D_ and *r*_A_ are the radii of the spherical cavities enclosing donor and acceptor (which are assumed to be spherical) and *R* is the distance between the centers of the cavities, *i.e.*, the ET distance.

### FOB-SH Nonadiabatic Molecular Dynamics

In FOB-SH, the valence (or conduction) band of OSs is described by the following Hamiltonian^17,35^

22 $$H=\sum_{k}^{N_{\mathrm{mol}}}\epsilon_{k}\left|\phi_{k}\right\rangle \left\langle \phi_{k}\right|+\sum_{k\neq l}^{N_{\mathrm{mol}}}H_{\mathrm{kl}}\left|\phi_{k}\right\rangle \left\langle \phi_{l}\right|$$

where, ϕ_*k*_ = ϕ_*k*_(**R**(*t*)) is the orthogonalized HOMO (LUMO) on a given molecule *k* for hole (electron) transport, **R**(*t*) are the time-dependent nuclear coordinates, ϵ_*k*_ = ϵ_*k*_(**R**(*t*)) is the site energy, that is, the potential energy of the state with the excess charge (hole or electron) located at site *k* and *H*_*kl*_ = *H*_*kl*_(**R**(*t*)) is the electronic coupling between ϕ_*k*_ and ϕ_*l*_. All Hamiltonian matrix elements, i.e., site energies and couplings, depend on the nuclear coordinates which, in turn, depend on time, **R =** **R**(*t*) as determined by the nuclear dynamics. The *N*_mol_ site energies ϵ_*k*_ are calculated using parametrized classical FF where molecule *k* is in the charged state and all other molecules *l* ≠ *k* in the neutral state. This constitutes a computational bottleneck when electrostatics is included, that we aim to remove using the DSF addition-subtraction scheme introduced above.

In the FOB-SH approach, the charge is described by a time-dependent 1-particle wave function, Ψ­(*t*), expanded in the same basis that is used to represent the Hamiltonian eq 22

23 $$\Psi \left(t\right)=\sum_{k=1}^{N_{\mathrm{mol}}}u_{k}\left(t\right)\phi_{k}\left(R\left(t\right)\right)$$

where *u*_*k*_ are the expansion coefficients. Insertion of eq 23 in the time-dependent Schrödinger equation gives the time-evolution of the charge carrier wave function in the valence (conduction) band

24 $$i\hbar {\mathrm{u̇}}_{k}\left(t\right)=\sum_{l=1}^{N_{\mathrm{mol}}}u_{l}\left(t\right)\left(H_{\mathrm{kl}}\left(R\left(t\right)\right)-i\hbar d_{\mathrm{kl}}\left(R\left(t\right)\right)\right)$$

where *d*_*kl*_ = ⟨ϕ_*k*_|ϕ̇_*l*_⟩ are the nonadiabatic coupling elements. The nuclear degrees of freedom are propagated on one of the potential energy surfaces (PES) obtained by diagonalizing the Hamiltonian eq 22 and denoted as “active surface”. While nuclear motion couples to the motion of the excess charge via the dependences on **R**(*t*) in eq 24, feedback from the excess charge to the nuclear motion is accounted for by transitions of the nuclear dynamics (“hops”) from the PES of the active surface to the PES of another eigenstate *j* using Tully’s surface hopping probability.^36^

In the application of FOB-SH presented further below, the delocalization of the charge carrier wave function is described by the inverse participation ratio

25 $$\mathrm{IPR}\left(t\right)=\frac{1}{N_{\mathrm{traj}}}\sum_{n=1}^{N_{\mathrm{traj}}}\frac{1}{\sum_{k=1}^{N_{\mathrm{mol}}}|u_{k,n}\left(t\right){|}^{4}}$$

where *N*_traj_ is the number of surface hopping trajectories and *u*_*k*,*n*_ the wave function expansion coefficient *u*_*k*_ in trajectory *n*. The IPR provides a measure for the number of molecules over which the charge carrier wave function Ψ­(*t*) is delocalized.

The charge mobility tensor is obtained from the mean square displacement (MSD) of the charge carrier wave function

26 $$\mu_{\alpha \beta }=\frac{eD_{\alpha \beta }}{k_{B}T}$$

where α­(β) represent *x*, *y*, *z* Cartesian coordinates, *e* is the elementary charge, *k*_B_ the Boltzmann constant and *T* the temperature. The diffusion tensor components, *D*_αβ_, can be obtained as time derivative of the mean squared displacement along the Cartesian components (MSD_αβ_)­

27 $$D_{\alpha \beta }=\frac{1}{2}\lim_{t\to \infty }\frac{d{\mathrm{MSD}}_{\alpha \beta }\left(t\right)}{dt}$$

where,

28 MSDαβ(t)=1Ntraj∑n=1Ntraj⟨Ψ(t)|(α−α0)(β−β0)|Ψ(t)⟩=1Ntraj∑n=1Ntraj∑k=1NmolPk,n(t)(αk,n−α0)(βk,n−β0)

in which α­(β) is the Cartesian coordinate operator of the charge with respect to the starting position (α_0_(β_0_)), *P*_*k*,*n*_(*t*) = |*u*_*k*,*n*_|^2^ is the time dependent charge population on a given site *k* obtained by solving eq 24 for trajectory *n*.

### Simulation Details

In this work calculations on crystalline ANT are carried out with three different types of force fields, all of which are based on the GAFF force field: (i) a nonpolarizable force field with fixed unscaled point charges (ii) an implicitly polarizable force field with fixed scaled point charges (iii) a polarizable force field with fixed unscaled point charges and atomic polarizabilities.

#### Nonpolarizable Force Field

The neutral state of a single anthracene molecule is modeled by the standard GAFF force field parameters.^37^ The force field equilibrium bond lengths in the neutral state (superscript “n”) are denoted **r** ^n,FF^ and the corresponding atomic point charges *q*_*i*_^n,FF^. To model the charged cationic state of anthracene (superscript “c”), we first obtain a new set of force field charges via *q*_*i*_^c,FF^ = *q*_*i*_^n,FF^ + Δ*q*_*i*_^DFT^, where Δ*q*_*i*_^DFT^ = *q*_*i*_^c,DFT^ – *q*_i_^n,DFT^ and *q*_*i*_^c,DFT^ and *q*_*i*_^n,DFT^ are the electrostatic potential-derived (ESP) atomic charges in the charged and neutral state of anthracene in the respective equilibrium geometries obtained from DFT calculations in vacuum. Here, symmetrized ESP charges were obtained using the Merz–Kollman scheme to fit the electrostatic potential.^38^ Then the force field equilibrium bond lengths for the charged state are adjusted by suitably scaling the corresponding DFT bond displacements, Δ**r** ^DFT^ = **r** ^c,DFT^ – **r** ^n,DFT^, and adding them to the force field equilibrium bond lengths for the neutral state, **r** ^c,FF^ = **r** ^n,FF^ + βΔ**r** ^DFT^. The scaling constant β is adjusted until the 4-point reorganization energy for hole self-exchange between two anthracene molecules in vacuum obtained from the force field matches the value obtained from DFT calculations (eq 19), giving β = 0.88.^16^ This reorganization energy is then defined to be the inner-sphere or internal reorganization energy for hole transfer in the crystalline system, λ_i_^4p^. All other force field parameters for the charged state remain the same as for the neutral state. DFT calculations for the reorganization energy were carried out with the B3LYP functional using the 6–311g­(d) basis set. We checked that 6–311g­(d) and 6–31g­(d,p) basis sets give similar λ_i_^4p^ and sets of charges (maximum difference 0.09 e). The sets of atomic charges *q*_*i*_^n,DFT^, *q*_*i*_^c,DFT^, *q*_*i*_^n,FF^, and *q*_*i*_^c,FF^ are summarized in Table S1, and the equilibrium bond lengths **r** ^n,DFT^, **r** ^c,DFT^, **r** ^n,FF^, and **r** ^c,FF^ in Table S2. We note in passing that, although this strategy is largely suitable for stiff molecules, it can also be generalized to more flexible molecules, where the charge state involves conformational changes in angles and dihedrals, by parametrizing the force field using more refined procedures. For instance, semiautomated strategies that employ energies, gradients, and Hessians from electronic-structure calculations can be used to obtain tailored, quantum-mechanically derived force fields for different kinds of molecules.^39,40^

#### Implicit Polarizable Force Field via Charge Scaling

Force fields with fixed atomic point charges do not account for electronic polarization and dielectric screening. This shortcoming is well documented in the charge transfer simulation literature.^41–45^ It leads to overestimated outer-sphere or external reorganization energy and activation energies for electron transfer, as well as to exaggerated thermal fluctuations of site energies. The latter issue is particularly problematic in nonadiabatic dynamics simulations of charge transport. While the problem can in principle be addressed by performing FOB-SH simulations with an electronically polarizable force field, the associated computational cost is prohibitive, especially when performing nonadiabatic MD on large nanoscale systems. Here, we adopt a simple charge-scaling scheme in which the fixed atomic charges obtained as explained above are scaled by a scaling constant γ, *q*_*i*_^n,FF^ → *q̃*_*i*_^n,FF^ = γ*q*_*i*_^n,FF^ and *q*_*i*_^c,FF^ → *q̃*_*i*_^c,FF^ = γ*q*_*i*_^n,FF^ + Δ*q*_*i*_^DFT^. The scaling constant is iteratively optimized until the total reorganization energy for hole transfer between two neighboring molecules in an anthracene crystal as obtained from MD simulation, λ^st^ (eq 15), matches the total reorganization energy obtained from MD simulation with the polarizable force field detailed further below. The optimal value was determined to be γ = 0.80. In principle, the scaling of the charges would require a readjustment of the bond displacements to maintain λ_i_^4p^ from DFT calculations. However, we found that the required readjustments are so small that they can be neglected. All force field parameters other than the scaled charges, *i.e.*, bonded and van-der-Waals interactions, are the same as for the nonpolarizable force field above.

#### Polarizable Force Field

The polarizable force field employed the same bonded interactions, point charges and van-der-Waals interactions as the nonpolarizable force field above, i.e., **r** ^n,FF^, *q*_*i*_^n, FF^ and **r** ^c,FF^, *q*_*i*_^c,FF^ for neutral and charged anthracene, respectively. Electronic polarizability was accounted for by induced atomic point dipoles with isotropic atomic polarizabilities taken from the Amoeba polarizable force field^46^ (α_H_ = 0.496 Å^3^, α_C_ = 1.334 Å^3^). These values represent refined versions of Thole’s isotropic polarizabilities^47^ derived from a large set of DFT calculations on representative molecules. The induced dipoles were iterated until the change in each dipole component was less than 10^–6^ a.u. We used the induced dipole implementation for periodic systems in CP2K,^48^ where the polarization model allows for the damping of the short-range induced dipoles interaction to prevent overpolarization using the Tang-Toennies damping functions. These functions are different from standard Thole’s damping functions used in the Amoeba model. Since no damping paramaters are available for anthracene or related systems, we have tested the effect of application of these functions applying typical short-range damping lengths (0.2–0.5 Å), and found that the total 4-point reorganization energy (λ^4p^) is insensitive to their inclusion. As the polarizable simulations converge well without the damping functions, all polarizable MD simulations were performed without additional damping.

#### MD Simulations for Testing DSF vs Ewald Summation

We used the same simulation cell as in ref 17 containing 4 × 4 × 4 unit cells (128 molecules) and run a MD simulation with the nonpolarizable force field with all molecules modeled in their charge-neutral state. The simulation was run for 1 ns and carried out in the NVT ensemble at 300 K using a Nose-Hoover thermostat applying a time step of 1 fs. Electrostatic forces in the MD run were calculated using Ewald summation with a damping constant for the real-space sum set to the default value, η = 0.35 Å^–1^, and the real-space interactions truncated at a cutoff value *R*_cut_^EW^ = 9.6 Å. We verified that with this setting of the reciprocal-space part of the Ewald summation is sufficiently converged for a maximum reciprocal-space vector magnitude of 0.6 Å^–1^, see Figure S2, though in all Ewald calculations we used 1.0 Å^–1^. Then, 1000 snapshots were extracted from this trajectory at an equidistant spacing of 1 ps and the DSF electrostatic energies and forces calculated on these snapshots for different values of the cutoff radius *R*_cut_ and damping constant α and compared to the results from Ewald summation.

#### MD Simulations for Calculation of Reorganization Energies

A snapshot from a 4 × 4 × 4 supercell of ANT equilibrated to 300 K was taken and equilibrated for 0.5 ns in the NVE ensemble for the following force fields (i) nonpolarizable force field with Ewald summation of electrostatic interactions (ii) nonpolarizable force field with DSF electrostatics (iii)–(v) implicit polarizable force field with charge scaling factors γ = 0.7, 0.75, and 0.80 using DSF electrostatics and (vi) polarizable force field with Ewald summation of fixed charge and induced dipole interactions. In these MD simulations one anthracene molecule (denoted molecule 1) was treated in the cationic charge state and all other molecules in the charge-neutral state, corresponding to the initial hole transfer state (A), with potential energy *E*_A_. The simulations were continued for 0.5 ns and 500 snapshots were extracted from these trajectories in equidistant spacings of 1 ps and used for the calculation of the potential energy for the final hole transfer state (B), *E*_B_. In the calculation of *E*_B_, molecule 1 was treated in the charge-neutral state and a next-nearest neighbor molecule (molecule 2) in the cationic charge state. Molecule 2 was chosen to be along the *b*-crystallographic direction forming a “P” (parallel) dimer with molecule 1 or along the *a*-crystallographic direction forming a “T” (T-shaped) dimer with molecule 1. The difference between *E*_B_ and *E*_A_ is the vertical energy gap eq 16 for hole transfer between the P or T oriented nearest neighbors. The corresponding reorganization energies eqs 15 and 17 were obtained from the vertical energy gap averaged over the 500 snapshots. Finite size effects for reorganization energy were investigated using the polarizable force field by equilibrating additional supercells comprised of 2 × 2 × 2, 6 × 6 × 6, and 8 × 8 × 8 unit cells. We found that the total reorganization energy (λ^4p^) was converged for the 4 × 4 × 4 system (see Table S3), and this supercell was subsequently used for the nonadiabatic MD simulation of charge transport as detailed below.

#### Nonadiabatic MD Simulations

FOB-SH simulations of hole transport in the conductive *a*-*b* plane of crystalline ANT were performed following a similar protocol as detailed in ref 17. A large supercell containing 12 × 20 × 2 unit cells (960 molecules) was equilibrated to 300 K, where all molecules were treated in the charge-neutral state. A 2D slab of 378 molecules within the *a*-*b* plane was selected and treated as electronically active. That is, each molecule *k* within the electronically active regions contributes their highest occupied molecular orbital (HOMO), ϕ_*k*_, to the expansion of the carrier wave function eq 23 and Hamiltonian eq 22. All other molecules of the supercell were treated as electronically inactive and interacted with the electronically active region via nonbonded and electrostatic interactions. A similar simulation cell was prepared for a 1D FOB-SH simulation by treating a stack of 36 molecules along the *b*-crystallographic direction as electronically active.^15^ The initial hole carrier wave function was chosen to be localized on a single molecule *i* within the electronically active region, Ψ(0) = ϕ_*i*_ and propagated in time according to eq 24 in the NVE ensemble. FOB-SH simulations were carried out with site energies ϵ_*k*_ and corresponding forces calculated with the nonpolarizable force field and with the implicit polarizable force field applying a charge scaling constant γ = 0.80 and employing the new DSF electrostatics addition-subtraction scheme. Here, the DSF electrostatic interactions between all atoms within the electronically active region, within the electronically inactive region, and between the electronically active and inactive region were included. FOB-SH simulations were also carried out with all electrostatic interactions excluded. All FOB-SH simulations applied a decoherence correction, state-tracking for detection of trivial crossings, a projection algorithm for removal of decoherence correction-induced artificial long-range charge transfer, and adjustment of the velocities in the direction of the nonadiabatic coupling vector in case of a successful surface hop.^14,15^ The nuclear time step, Δ*t*, was set to 0.1 fs, a value previously found to strike a good balance between accuracy and computational cost.^17^ The electronic time step for integration of eq 24 using the Runge–Kutta algorithm to fourth order was δ*t* = Δ*t*/5. For each simulation at least 600 FOB-SH trajectories of length 1 ps were run over which the IPR, MSD and charge mobility were averaged.

## Results

### DSF vs Ewald Summation

The DSF energy eq 6 and the corresponding forces eq 14 were implemented in the CP2K package.^49^ In the following we validate our DSF implementation against exact Ewald summation on snapshots taken from a 1 ns finite temperature trajectory run for a supercell of crystalline ANT where all anthracene molecules were treated in their charge-neutral state (see Results section for further simulation datails). The results are summarized in Figure 2. In panels (a,b) we report the mean unsigned error (MUE) and the maximum (max) error of the electrostatic DSF energy with respect to the Ewald energy, normalized by the mean unsigned fluctuation (MUF) of Ewald energies (eqs S1 and S2), for different values of the cutoff radius *R*_cut_ and damping constant α defining the DSF electrostatic interaction energy (eq 7). The corresponding errors for the electrostatic forces are reported in Figure 2(c,d). We find that the error in energies and forces initially sharply decreases and then saturates with increasing cutoff radius *R*_cut_ for all values of α. The smallest error, <1%, is obtained at the largest cutoff investigated, *R*_cut_ = 35 Å for α = 0.05. This shows that with a suitable choice of α the electrostatic energies and forces approach very closely the results from exact Ewald summation. However, a large cutoff radius of 35 Å is computationally expensive. We find that a reasonable compromise between accuracy and computational cost is reached with *R*_cut_ = 16 Å and α = 0.09 Å^–1^, where the average error in energy and forces is <5% and the max error <30%. These values are used in all DSF calculations presented further below. As an aside, we note that in the limit α → 0 the damping function is completely removed, and the DSF potential reduces to a purely shifted Coulomb potential with a finite cutoff,^8^ which is equivalent to the undamped shifted-force (SF) method, originally proposed by Wolf et al.^6^ In this situation, there is slower convergence with respect to *R*_cut_ and larger cutoff radii are required to achieve Ewald-like accuracy, at the expense of increased calculation time.

**Figure 2:**
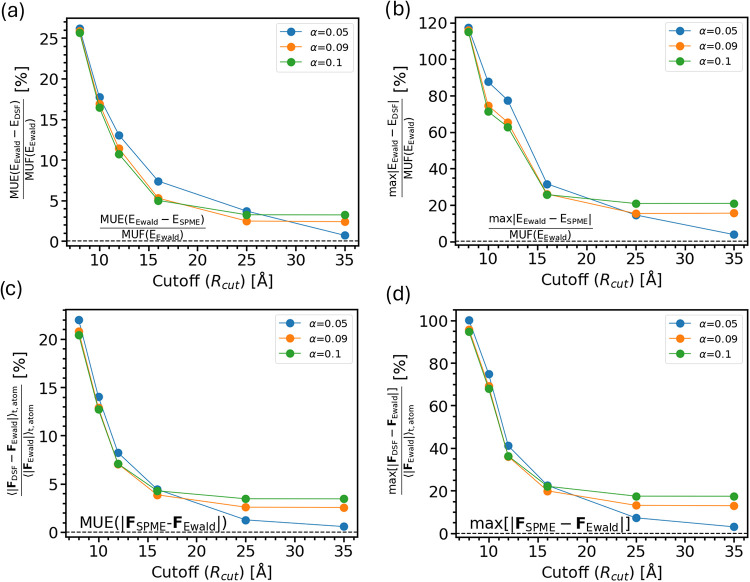
DSF electrostatic energies and forces benchmarked against Ewald summation. (a) Mean unsigned error (MUE) and (b) maximum error of the DSF electrostatic energy for an ensemble of configurations taken from an MD trajectory of an ANT crystal at 300 K. The MD simulation was run with Ewald electrostatics and the DSF energy and forces were recalculated along that trajectory. The errors are normalized by the Mean Unsigned Fluctuation, MUF (Ewald), and shown as a function of the DSF real-space cutoff *R*_cut_ and for different values of the damping coefficient α as indicated by the different colors. The DSF interaction potential is defined in eq 7, the unit of α is Å ^–1^. To put these errors in perspective, the electrostatic energy difference between Ewald and SPME calculations are indicated by horizontal dashed lines (0.014% and 0.056% in panels (a, b), respectively). Panels (c, d) show the same error metrics as in (a, b), but for the unsigned absolute differences between DSF and Ewald electrostatic forces averaged over all atoms and all time steps, with deviations between Ewald and SPME again indicated by dashed horizontal lines.

Next we investigate the accuracy of the DSF method in describing the thermal fluctuations of electrostatic energy and forces. We consider the same ensemble of snapshots used above and plot the electrostatic energy relative to the mean value as a function of time in Figure 3(a) and the related probability distribution in Figure 3(b). Both DSF and Ewald give very similar energy fluctuations, with a standard deviation σ of 134 and 138 meV, respectively. Note that the difference between DSF and Ewald energy remains always below ≈20 meV, confirming the good accuracy of DSF compared to Ewald when using the optimal parameters for *R*_cut_ and α. In Figure 3(c,d), we report a similar comparison for the force component of an arbitrarily selected carbon atom of the system. Here again, the agreement with Ewald is excellent. The difference between the DSF and Ewald force remains below a maximum difference <0.025 mHa bohr^–1^ at all times, which is an order of magnitude lower than typical force convergence thresholds in geometry optimizations.

**Figure 3:**
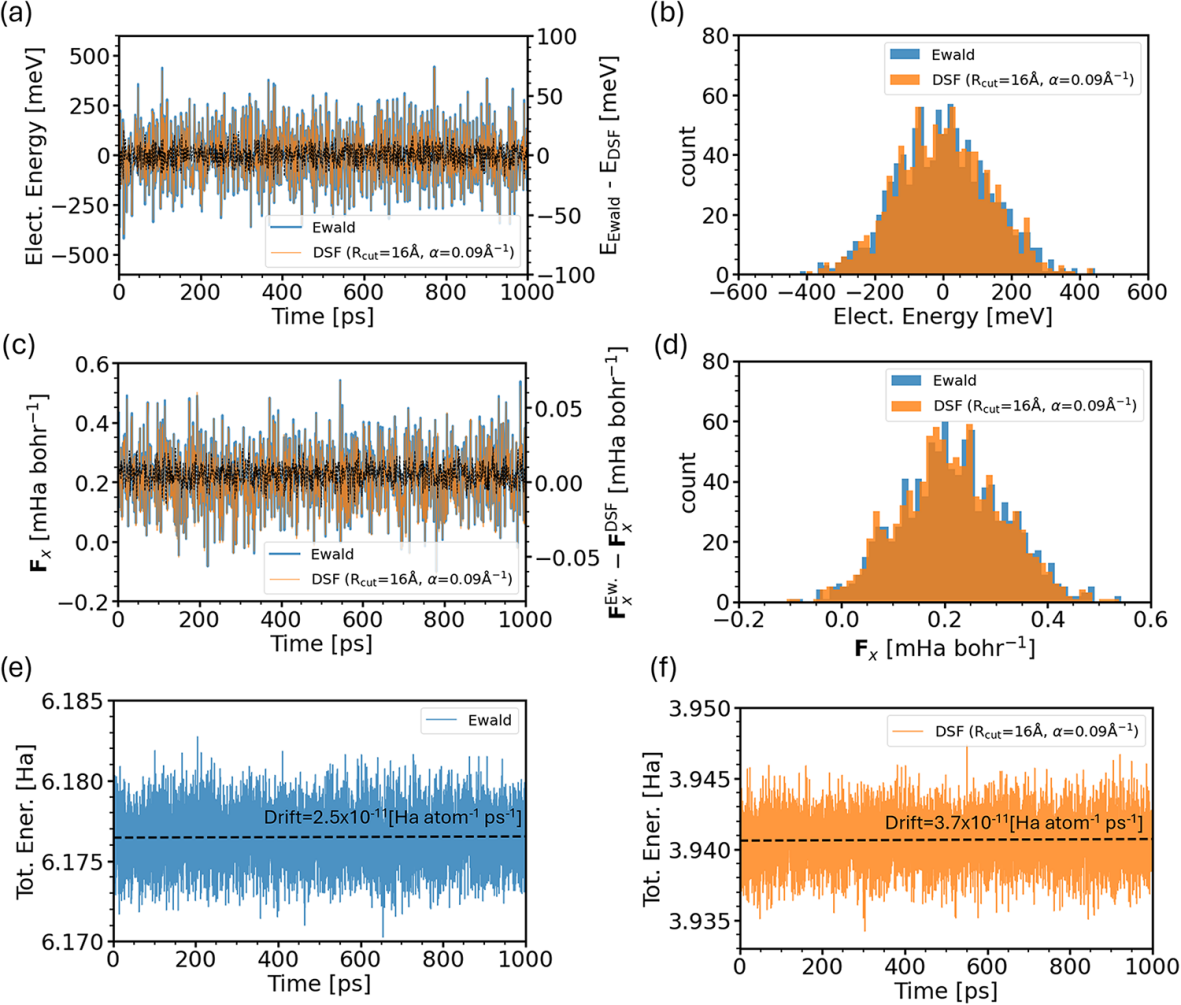
Thermal fluctuations of electrostatic energy and total energy conservation in DSF and Ewald summation. (a) DSF (orange, *R*_cut_ = 16 Å, α = 0.09 Å ^–1^) and Ewald electrostatic energy (blue) as a function of MD simulation time for an ANT crystal at 300 K, with mean values set equal to zero. The same data were used for the error analysis in Figure 2. The difference between DSF and Ewald electrostatic energy is shown in black lines (*y*-axis to the right). (b) Distributions of DSF (orange) and Ewald electrostatic energies (blue) of the time series shown in panel (a). Panel (c) shows the DSF (orange) and Ewald electrostatic force (blue) for an arbitrarily chosen atom along the *a*-crystallographic direction as a function of simulation time, and panel (d) shows the corresponding force distributions. Panels (e, f) report the total energy along a 1 ns NVE trajectory performed with DSF and Ewald methods, respectively. Dashed black lines represent best linear fits to the total energy drift.

Finally, we analyze the total energy conservation of our DSF implementation. To this end, we took an equilibrated snapshot from the above trajectory and ran additional classical MD simulation for 1 ns in the NVE ensemble using DSF electrostatics, and in a separate run, exact Ewald summation. We then computed the total energy as a function of time as shown in Figure 3(e,f). The energy drift in both simulations remains on the order of 10^–11^ Ha atom^–1^ ps^–1^, and the relative fluctuations in the total energy (σ­(*E*_tot_)/⟨*E*_tot_⟩) is 0.04% and 0.03% for DSF and Ewald, respectively, implying that the DSF algorithm is correctly implemented.

### Scaling with System Size and Speed-Up

To investigate the scaling of the computational cost of DSF calculations with respect to system size we consider a series of supercells of increasing size, 5 × 5 × *N*, where *N* = 1, 2, 3, 5, 7, 10, 15, 20 (corresponding to *N*_mol_ = 50–1000 molecules), and calculate the electrostatic energy and forces for a single charge-neutral state using DSF, exact Ewald summation and SPME. The results are shown in Figure 4. As expected, the DSF calculations scale linearly with system size (slope = 1.0 on a log (time)–log (system size) plot), similarly as SPME (slope = 0.9). Conversely, exact Ewald summation scales unfavorably (slope = 2.8, which might be lowered to a value closer to 2 through better balancing of real and reciprocal space parts via tuning of η). We note that for a single charge-state the DSF calculation is slower than for SPME due to the relatively large optimal real-space cutoff required in DSF (*R*_cut_ = 16 Å), but faster than exact Ewald summation for all except the smallest system due to the linear scaling.

**Figure 4:**
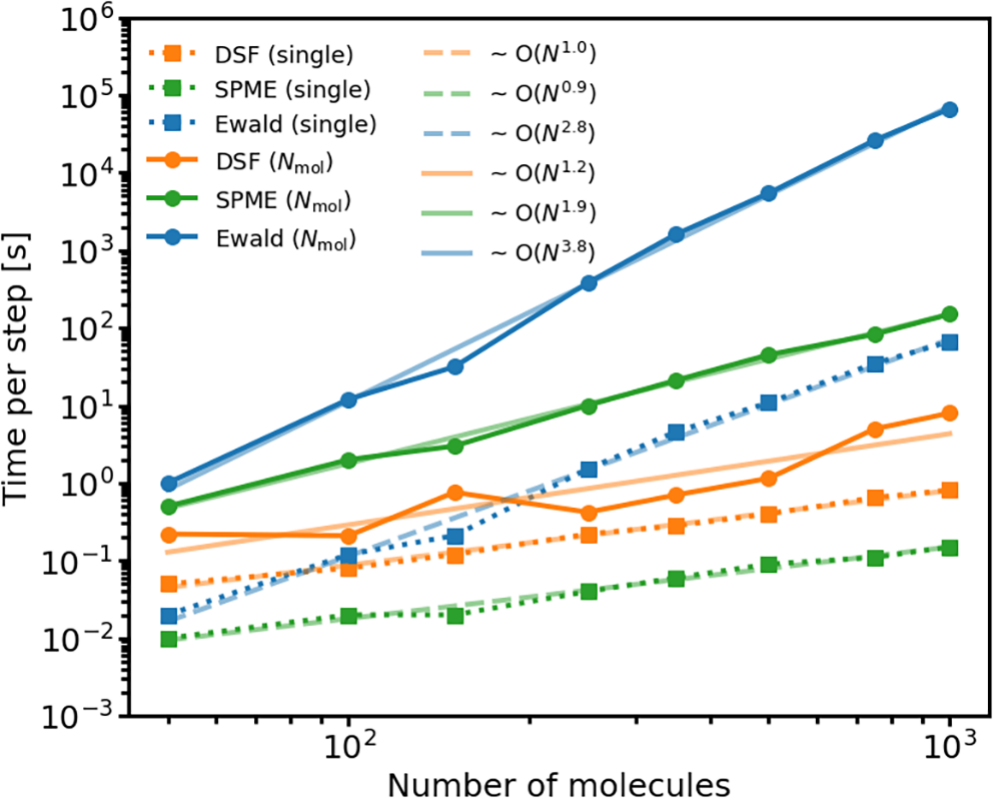
Timing of MD simulation with DSF and Ewald electrostatics as a function of system size. The CPU time per MD step is shown as a function of the number of anthracene molecules in a periodically replicated supercell (*N*_mol_) using DSF electrostatics (data in orange), Ewald summation (data in blue) and SPME (data in green). The timings for the calculation of energies and forces for a single charge state are shown in dotted lines and for the *N*_mol_ different charge states in thick solid lines. For DSF electrostatics, the calculation of the *N*_mol_ different charge states were carried out with the addition-subtraction scheme, whereas for Ewald and SPME electrostatics, the timings for a single charge state were simply multiplied by a factor *N*_mol_. Best linear fits of the timing data are shown in thin solid lines with slopes that are numerically equal to the exponents reported in the figure legend. Notice that the computational effort for the calculation of all *N*_mol_ different charge states scales linearly with system size when DSF electrostatics is used in combination with the addition-subtraction scheme. The MD simulations were run on a single CPU (Intel Core i7–14700).

Next we consider the timing for the calculation of electrostatic energy and forces for the *N*_mol_ different charged states of the system as a function of the supercell size. In each of the *N*_mol_ charged states one of the ANT molecules is in the cationic state (equilibrium bond lengths **r** ^c,FF^ and atomic charges *q*_*i*_^c,FF^) while all other molecules are in charge-neutral state (equilibrium bond lengths **r** ^n,FF^ and atomic charges *q*_*i*_^n,FF^). Without the addition-subtraction scheme the computational overhead relative to the calculation for a single charge state trivially increases by a factor of *N*_mol_ charge states. This is illustrated in Figure 4, where the slope for both SPME and Ewald increases from about 0.9 for a single charge state, to 1.9 for *N*_mol_ in SPME and from 2.8 to 3.8 in Ewald. We note in passing that in SPME the timings for a single charge state in Figure 4 were simply multiplied by a factor *N*_mol_, which provides an upper estimate of the computational cost. This is because, in an efficient implementation of SPME for *N*_mol_ charge states, the interpolation of atomic positions on the FFT grid only needs to be performed once, rather than *N*_mol_ times, thereby resulting in some cost savings.

Applying the addition-subtraction scheme only to the real-space parts of SPME and Ewald does not change this situation. The bottleneck is the reciprocal space sum which cannot be straightforwardly optimized using the addition-subtraction scheme. By contrast, using DSF in combination with the addition-subtraction scheme, the calculation of energies and forces for all *N*_mol_ charge states still scales linearly with system size (slope = 1.2); merely the prefactor or, on a log–log plot, the intercept changes by about a factor of 5.

As we will see below, in order to converge charge mobility with FOB-SH simulations, one typically needs system sizes of several hundred molecules. Here, we will use supercells containing 378 ANT molecules. According to Figure 4 the calculation of all *N*_mol_ = 378 charge states at a given MD time step would take more than 1000 s with brute force Ewald summation and about 20 s with SPME, whereas for DSF in combination with the addition-subtraction scheme it takes only about 0.5 s. It is evident from the above timings that brute-force Ewald or even SPME calculations of the *N*_mol_ charge states quickly become impractical and that DSF in combination with the addition-subtraction scheme makes these calculations feasible in a linear scaling fashion with a reasonably small prefactor.

### Reorganization Energies

The total reorganization energy is calculated for hole transfer between two nearest neighbor molecules in the ANT crystal that have a “P” (parallel) or “T” (T-shaped) orientation, closely aligning with the *b*- and *a*- crystallographic directions. It includes both internal and external contributions and is calculated in three different ways: at 0 K according to the 4-point scheme, λ^4p^ eq 19, using geometry optimization of the supercell, at 300 K from the mean energy gap, λ^st^ eq 15, and from the fluctuations of the energy gap, λ^var.^ eq 17, using MD simulations. In each of the three methods the calculations were carried out with a nonpolarizable force field using DSF or Ewald summation for the electrostatics, with an implicitly polarizable force field that uses scaled fixed charges and DSF electrostatics, and with a polarizable force field with induced point dipoles where all electrostatic interactions were calculated with Ewald summation. We refer to Section 3 for details of the force field parametrization and for simulation details. The results are summarized in Table 1.

**Table 1:** Reorganization Energies for Hole Transfer in Crystalline ANT for Different Treatments of Electrostatic Interactions

Electrostatics	polarizability	γ^a^	λ^4p^ ^b^ (meV)	λ^st^ ^c^ (meV)	λ^var.^ ^d^ (meV)
P dimer (*a*-direction)					
Ewald	none	1	181	219 ± 7	220
DSF	none	1	181	205 ± 12	227
DSF	implicit	0.80	166	188 ± 11	187
DSF	implicit	0.75	164	186 ± 10	195
DSF	implicit	0.70	160	179 ± 10	180
Ewald	induced dipole	1	173	190 ± 8	180

T dimer (*b*-direction)					
Ewald	none	1	185	208 ± 6	199
DSF	none	1	183	218 ± 12	230
DSF	implicit	0.80	167	191 ± 9	164
DSF	implicit	0.75	165	179 ± 10	179
DSF	implicit	0.70	161	178 ± 9	170
Ewald	induced dipole	1	176	187 ± 8	192

a Charge scaling constant, see Results section.

b Reorganization energy from 4-point Scheme eq 19 at 0K.

c Reorganization energy from the difference in mean energy gaps (Stokes shift), eq 15, obtained from MD simulation at 300 K. Statistical uncertainties obtained by block averaging are given.

d Reorganization energy from the variance in the energy gap, eq 17, obtained from MD simulation at 300 K.

We find that across all three definitions of reorganization energy, nonpolarizable MD with DSF or Ewald electrostatics agree very closely (compare “DSF (γ = 1)” and “Ewald”). The difference between DSF and Ewald for λ^4p^ is negligible and for λ^st^ < 7%. The latter is within the statistical uncertainty of the MD estimate for λ^st^. Interestingly, we find that the finite temperature leads to a small increase in reorganization energy judging from the difference in λ^4p^ and λ^st^. Moreover, the reorganization energy from the Stokes shift, λ^st^, and from the fluctuations, λ^var.^, are fairly similar implying that the system is well within the linear response regime, where the two definitions of reorganization energy are exactly equivalent.

Our best estimate for total reorganization energy is provided by the electronically polarizable MD simulations giving values of λ^st^ = 190 ± 8 and 187 ± 8 meV for the P and T orientation, respectively. Subtracting the internal contribution λ_i_^4p^ = 142 meV from these values gives an external contribution λ_o_^st^ = 48 and 45 meV. (Note, here we have assumed that λ_i_^4p^ ≈ λ_i_^st^, i.e., we have neglected the small temperature dependence of the inner-sphere contribution.) For the P and T orientation respectively, nonpolarizable MD simulation gives values of: λ_o_^st^ = 77 ± 7 and 66 ± 6 meV using Ewald electrostatics and 63 ± 12 and 76 ± 12 meV using DSF electrostatics. Hence, electronic polarization leads to a decrease in external reorganization energy by a factor *p* ^MD^ = λ_o_^st^(nonpol)/λ_o_^st^(pol) = 1.61 and 1.46 along P and T direction for Ewald electrostatics and by factors 1.33 and 1.70 for DSF electrostatics. Interestingly, similar values for *p* ^MD^ have been reported before for electron transfer in protein aqueous solutions.^33^

The effect of electronic polarization on external reorganization energy may also be estimated from Marcus’ continuum electrostatics expression eq 21, as the ratio of Pekar factors, *p* ^c^, with the optical dielectric constant set to unity (corresponding to the electronically nonpolarizable force field) or to the experimental value (corresponding to the (ideal) polarizable force field), *p* ^c^ = (1 – 1/ϵ_s_)/(1/ϵ_op_ – 1/ϵ_s_). Inserting the experimental values of optical and static dielectric constants for anthracene, ϵ_op_ = 2.6 (measured in the thin-film^50^) and ϵ_s_ = 3.2 (approximated as the mean value of three principal components of static dielectric tensor of anthracene crystal^51^), we obtain *p* ^c^ = 9.5. While part of the deviation from the MD result above may be related to the fact that eq 21 was derived for spherical ions, not planar molecules like ANT, the very strong overestimation of *p* ^c^ compared to *p* ^MD^ points to intrinsic limitations of the continuum model that leads to a too strong dependence of reorganization energy on the optical dielectric constant. Similar observations were made some time ago.^43^ Instead we observe that the above values for *p* ^MD^ averaged over the two crystallographic directions are close to ϵ_op_^1/2^ = 1.61. This suggests that the polarizable MD estimate of reorganization energy might be attained from nonpolarizable MD simulations if the charges are scaled by a factor γ = ϵ_op_^–1/2^ = 0.62.

Following this observation, we scale the fixed point charges and calculate the total reorganization energy in what we call implicitly polarizable MD simulations using DSF electrostatics. The results for different scaling values γ are summarized in Table 1. We again extract the external reorganization energy λ_o_^st^ by subtracting the internal contribution λ_i_^4p^ = 142 meV from the total reorganization energy. We find that λ_o_^st^(γ)< γλ_o_^st^(γ = 1) (Figure S13). This is because charge scaling has two effects. First, it leads to a scaled electrostatic field on the donor and acceptor molecules resulting in a scaled energy gap Δ*E* → γΔ*E* (eq 16) and a scaled external reorganization energy λ_o_^st^ → γλ_o_^st^. Yet, it also leads to a smaller structural reorganization of the molecules surrounding the donor–acceptor pair that lowers external reorganization energy further such that λ_o_^st^(γ)< γλ_o_^st^(γ = 1). Hence, the optimal charge scaling factor will be larger than what the above estimate γ = ϵ_op_^–1/2^ = 0.62 would suggest. Indeed, we find that a charge scaling factor γ = 0.80 gives reorganization energies λ^st^ along P and T directions, 188 ± 11 and 191 ± 9 meV, that are in very close agreement with the values from polarizable MD simulations stated above. This charge scaling value is used in FOB-SH simulation with DSF electrostatics for hole transport in ANT, that we will present in the following section.

### Charge Transport Simulations

We now turn to the simulation of hole transport in the conductive *a*-*b* plane of ANT and investigate the effect of electrostatics on hole wave function delocalization and mobility using FOB-SH nonadiabatic molecular dynamics in combination with our new DSF electrostatics addition-subtraction scheme. To this end, we carry out FOB-SH simulations where the electrostatic contribution to the site energies ϵ_*k*_ (eq 22) is calculated with the nonpolarizable force field and with the implicitly polarizable force field (with γ = 0.80) and compare the results to the ones where all electrostatic interactions are neglected (referred to as “no electrostatics”). For a brief explanation of the FOB-SH method and for simulation details we refer to Methods section.

The first important observation is that inclusion of electrostatics in FOB-SH has virtually no effect on the packing structure of ANT, the RMSD of the center of mass (COM) of the molecules remains the same when electrostatic interactions are excluded or included with unscaled or scaled charges, respectively (see also Figure S3). This implies that van-der-Waals interactions are sufficient to reproduce the packing structure of ANT accurately.

Next, we consider the IPR defined in eq 25, which is a measure for the number of molecules over which the hole wave function is delocalized. The delocalization of the wave function Ψ­(*t*) should not be confused with the charge localized nature of the quasi-diabatic states ϕ_*i*_: the latter states are merely used to expand Ψ­(*t*) according the eq 23. We find that the IPR averaged over all trajectories is about 5 if electrostatics is excluded. A representative snapshot of the hole wave function is shown in Figure 5a. Inclusion of electrostatics with implicit polarization leads to a decrease in IPR to about 3.5 and for nonpolarizable electrostatics to about 3.0, see Figure 5b. The reason for the decrease in IPR is that the electrostatic interactions lead to increased site energy fluctuations (also sometimes denoted diagonal electrostatic disorder) resulting in more localized bandstates (eigenstates) that the hole carrier wave function transiently occupies during the nonadiabatic dynamics. The increase in site energy fluctuations is consistent with the increased reorganization energy when external/electrostatic contribution is included due to eq 17. It is worth noting that in contrast to site energy, the electronic couplings (*H*_*kl*_ in eq 22) and their thermal fluctuations are not strongly affected by the presence of electrostatic interactions (see Figure S4) because the electrostatics of the environment does not give rise to significant changes in the relative orientation or distance between the molecules or to substantial polarization of the frontier orbitals which electronic couplings would be sensitive to.

**Figure 5:**
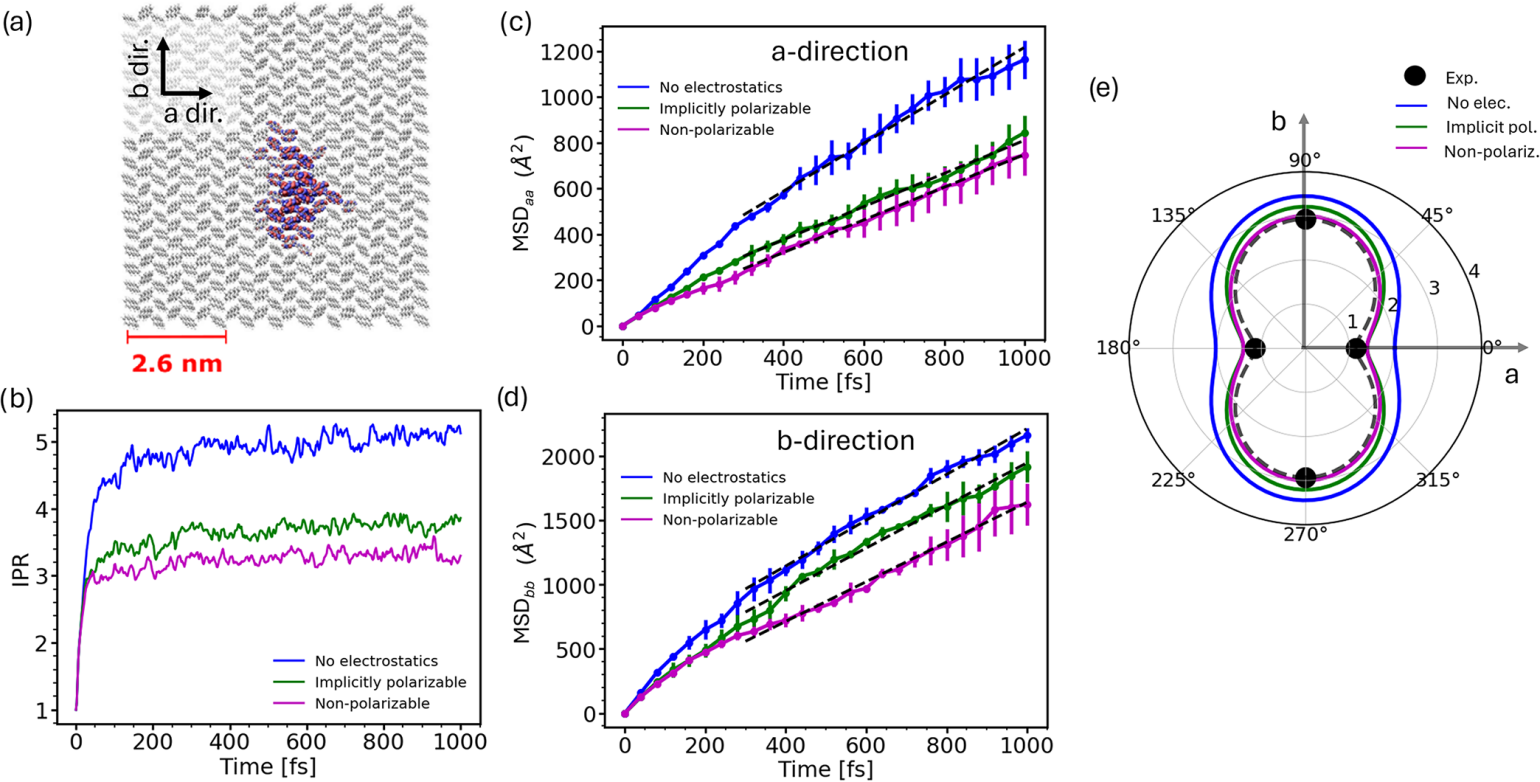
Hole transport in crystalline ANT from FOB-SH nonadiabatic dynamics. (a) Pictorial representation of the hole polaron, adapted from ref 17 Available under a Creative Commons CC-BY license. Copyright 2020 John Wiley and Sons. (b) IPR eq 25 and (c, d) components of the MSD tensor eq 28 along the *a*- and *b*-crystallographic directions for the hole wave function Ψ­(*t*) as a function of simulation time. The data were averaged over 600 FOB-SH trajectories. Electrostatic interactions in the site energy and force calculation are either excluded (data in blue) or included via the DSF addition-subtraction scheme employing the nonpolarizable force field with unscaled charges (data in magenta) or the implicitly polarizable force field using DSF electrostatics with scaled charges (green, γ = 0.80). Error bars in the MSDs were obtained by averaging over three blocks each containing at least 100 trajectories. Linear fits to the MSDs are shown in dashed lines and are used to obtain the hole mobilities via eq 26. (e) Hole mobility anisotropy plot in polar representation. Notice the improved agreement with experiment^52^ when electrostatics is included.

The MSD of the charge carrier wave function obtained from FOB-SH trajectories according to eq 28 is shown in Figure 5c,d. We find that the MSD increases linearly with time without and with electrostatics indicative of Einstein diffusion. Yet the slope is somewhat smaller when electrostatics is included, and this effect is more pronounced along the *a*- than along the *b*-crystallographic direction. Converting the slopes of the MSDs to mobilities using eq 26 we find that the mobilities decrease slightly upon inclusion of electrostatics with implicit polarization, from μ_*b*_ = 3.5 to 3.2 cm^2^ V^–1^ s^–1^ along the *b*-direction and from μ_*a*_ = 2.0 to 1.4 cm^2^ V^–1^ s^–1^ along the *a*-direction. Very similar values are obtained from nonpolarizable simulations implying that inclusion of implicit polarization via charge scaling is not very important in regards with the charge mobility of this system (see Table 2 for a summary of mobility values). Importantly, inclusion of electrostatics yields absolute mobility values that are now in very good agreement with experimental time-of-flight mobilities, reported to be 2.9 and 1.1 cm^2^ V^–1^ s^–1^ along the *b*- and *a*-direction, respectively.^52^ Moreover, the agreement in mobility anisotropy, μ_*b*_/μ_*a*_, is also improved, going up from 1.8 to 2.3 when electrostatics is included, compared to 2.6 in experiment.^52^ A corresponding polar plot of mobility in the *a*-*b* plane is shown in Figure 5e, clearly illustrating the improvement in the computed anisotropy compared to experiment when electrostatics is included.

**Table 2:** Hole Mobilities and IPR Values for Hole Transport in Crystalline Anthracene as Obtained from FOB-SH Simulations with Different Treatments of Electrostatic Interactions

		hole mobility (cm^2^ V^–1^ s^–1^)			IPR	
electrostatics	γ^a^	1D (*b*-dir.)^b^ ^,^ ^c^	2D (*b*-dir.)^d^ ^,^ ^e^	2D (*a*-dir.)^d^ ^,^ ^e^	1D^b^ ^,^ ^f^	2D^d^ ^,^ ^f^
none		1.9 ± 0.17	3.5 ± 0.02	2.0 ± 0.02	2.2 ± 0.03	5.0 ± 0.13
DSF^g^, nonpolarizable	1	1.1 ± 0.06	3.0 ± 0.06	1.4 ± 0.04	1.9 ± 0.03	3.3 ± 0.08
DSF^g^, implicitly pol.	0.80	1.2 ± 0.06	3.2 ± 0.03	1.4 ± 0.02	2.0 ± 0.03	3.7 ± 0.09
experimental^h^		2.9	2.9	1.1		

a Charge scaling constant, see Results section.

b FOB-SH simulation of hole transport in a reduced 1D model of crystalline ANT along the *b*-crystallographic direction.

c Mobility obtained from the linear fit of the MSD of the hole wave function Ψ­(*t*) according to eqs 26 and 27. The MSD is obtained from 1000 FOB-SH trajectories and the error bars are obtained by block-averaging over 5 blocks.

d FOB-SH simulation of hole transport in the *a*-*b* crystallographic plane of crystalline ANT.

e Mobility obtained from the linear fit of the MSD of the hole wave function (Ψ­(*t*)) shown in Figure 5(c,d) according to eqs 26 and 27. The MSD is obtained from 600 FOB-SH trajectories and the error bars are obtained by block-averaging over 3 blocks.

f Inverse participation ratio of the hole carrier wave function, eq 25. Error bars were obtained by block averaging the equilibrated region of the IPR.

g Employing the addition-subtraction scheme for electrostatic interactions, schematically illustrated in Figure 1.

h Ref 52.

Several works have established that delocalization of the charge carrier wave function enhances transport and increases mobility.^16,19,53,54^ Thus, the small decrease in both charge carrier delocalization and mobility upon inclusion of electrostatics is consistent with this trend. It has also been proposed on the basis of simulation^16,17^ and theory^53^ that in high mobility organic semiconductors charge transport occurs via a transient delocalization mechanism in which thermal fluctuations temporarily promote the charge (hole or excess electron) from an initial low-energy state to a more delocalized high-energy state, which then rapidly decays back to a low-energy state that is spatially displaced from the initial state. These transient delocalization events were shown to occur on the 100 fs time scale and result in large mean squared displacements (MSD) of the charge and in high mobility. We find that the same transient delocalization mechanism remains operative in ANT when electrostatics is included.

## Conclusion

We have developed an efficient computational scheme for calculating electrostatic energies and forces in molecular systems with multiple charge states. By combining the Damped Shifted Force (DSF) real-space summation method with the addition-subtraction scheme, we demonstrated that the computational overhead for calculation of *N*_mol_ charge states compared to a single charge state is reduced from *O*(*N*_mol_) to *O*(*N*_mol_^0^), that is just a constant size-independent factor, significantly improving scalability for large systems. Our implementation was benchmarked against full Ewald summation, showing very good agreement in energy and force accuracy at a reasonably small real-space cutoff distance as well as robust energy conservation in MD simulation.

The DSF addition-subtraction scheme is particularly useful, but not limited to, nonadiabatic molecular dynamics simulations that employ electronic Hamiltonians in a diabatic or site basis, eq 22. Here, the electrostatic contribution to the site energy ϵ_*k*_ of the *N*_mol_ different states needs to be computed every MD step. While brute-force Ewald summation is untractable for this task for practically relevant system sizes, even when SPME is used (see Figure 4), our DSF addition-subtraction scheme now enables such calculations.

In a proof-of-concept application, we used FOB-SH nonadiabatic dynamics supplemented with the DSF addition-subtraction scheme to simulate hole transport in crystalline ANT. We found that electrostatic effects lead to a small but not insignificant decrease in charge carrier delocalization and charge mobility that further improves the agreement with experimental time-of-flight mobility and mobility anisotropy.^52^ We expect the same to be the case for other acenes that we previously studied with FOB-SH, specifically rubrene^21^ and pentacene.^17^ One of the most important outcomes of our simulation is that the charge transport mechanism, transient delocalization, remains unchanged upon inclusion of electrostatics, and this is very likely to be the case also for the other apolar high-mobility organic crystals studied previously.^16–22^ We expect that electrostatic effects will be more pronounced in materials made of molecules that feature polar bonds or dipolar moieties such as nonfullerene acceptors.^20^ Here, electrostatics might tip the balance between transient delocalization and hopping transport; simulations on such materials will be carried out in future work.

The DSF addition-subtraction scheme can be readily applied to related processes, e.g., exciton transport and exciton dissociation, in nanoscale materials. We expect that the effect of electrostatics on the transport properties of neutral (Frenkel) excitons will be smaller than for charges because the coupling with the environment is merely in response to a change in the charge distribution (dipole and higher multipoles) rather than to a change in the charge of the molecule. Our computational framework enables a more realistic simulation of both charge and exciton dynamics in molecular systems at a small computational overhead, opening up applications to systems where electrostatic effects are deemed important, including nonfullerene acceptors, supramolecular aggregates formed in solution, biomolecules in water or complex matrices and biomaterials.

## Supplementary Material



## Data Availability

Additional data have been added to the Zenodo repository DOI: https://zenodo.org/records/17649929.

## Notes

Additional information on error quantification; computational details on simulation setup; tests and analysis of the convergence of electrostatic simulations, electronic parameters, and reorganization energies from molecular dynamics (PDF)

## ∥

STFC, UKRI, Hartree Centre, Daresbury Laboratory, Daresbury WA4 4AB, U.K

## ⊥

Institut für Chemie, Universität Kassel, Heinrich-Plett-Straße 40, 34132 Kassel, Germany.
