# Supplementary material for: Efficient Calculation of Electrostatic Energies for Large-Scale Nonadiabatic Molecular Dynamics in a Site Basis
Source: J Chem Theory Comput. 2025 Dec 23;22(1):151–65. doi: 10.1021/acs.jctc.5c01753 (PMC12805572; doi:10.1021/acs.jctc.5c01753)
Supplement: Supplementary file 1 [file ct5c01753_si_001.pdf]

Supporting Information: Efficient Calculation of Electrostatic Energies for Large-Scale Non-adiabatic Molecular Dynamics in a Site Basis

Samuele Giannini,[‖,†] Ljiljana Stojanovic,[§,‖,‡] Matthew Ellis,[‖,‡] Guido Falk von Rudorff,[¶,‡] and Jochen Blumberger[*,‡]

†*Department of Chemistry and Industrial Chemistry, University of Pisa, Via Giuseppe Moruzzi, 56124 Pisa, Italy*

‡*Department of Physics and Astronomy and Thomas Young Centre, University College London, London WC1E 6BT, UK*

E-mail: j.blumberger@ucl.ac.uk

[‖]Contributed equally to this work

[§]Present address: STFC, UKRI, Hartree Centre, Daresbury Laboratory, Daresbury, WA4 4AB, UK

[¶]Present address: Institut für Chemie, Universität Kassel, Heinrich-Plett-Straße 40, 34132 Kassel, Germany

Error quantification

In the following we define the error metrics used in Figure 1 of the main text. The Mean Unsigned Error (MUE) is defined as

$$\mathrm{MUE} = \frac{1}{N}\sum_{i=1}^{N}\left|y_i^{\mathrm{pred}} - y_i^{\mathrm{ref}}\right| \tag{S1}$$

where N is the total number of data points, y_i^{pred} denotes the predicted value for the i-th data point, and y_i^{ref} is the corresponding reference (true or experimental) value. The MUE thus measures the average absolute deviation between predictions and reference values. The Mean Unsigned Fluctuations Error (MUF) is defined as

$$\mathrm{MUF} = \frac{1}{N}\sum_{i=1}^{N}\left|f_i - \bar{f}\right| \tag{S2}$$

where f_i represents the instantaneous value of the fluctuating quantity and $\bar{f}$ is its arithmetic mean over all N samples. The MUF quantifies the average magnitude of fluctuations around the mean value, independently of their sign.

Additional simulations details

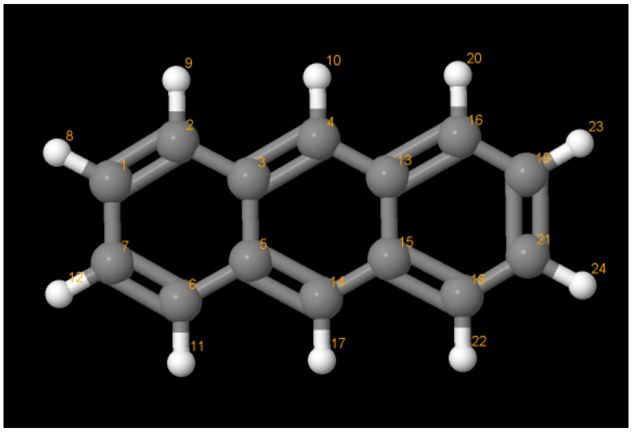

Figure S1: ANT molecule and atom indeces

Force field parameters

Table S1: Symmetry unique MK charges for the neutral and charge ANT molecules at B3LYP/6-31G(d,p) (elementary charge unit (e))

**Number**	**Atom**	neutral	charge
1	C	-0.1137	-0.0609
2	C	-0.2082	-0.1289
3	C	0.1741	0.1447
4	C	-0.3665	-0.2181
8	H	0.1197	0.1549
9	H	0.1292	0.1589
10	H	0.1641	0.1806

Table S2: Symmetry unique bond lengths for the neutral and charge optimized ANT molecules from QM (B3LYP/6-311G(d)) and FF (GAFF based)

		QM		FF (GAFF)	
Atom #1	Atom #2	$\mathbf{r}^{n,QM}$	$\mathbf{r}^{c,QM}$	$\mathbf{r}^{n,FF}$	$\mathbf{r}^{c,FF}$
5	3	1.443	1.439	1.490	1.490
3	2	1.429	1.412	1.398	1.383
4	3	1.398	1.408	1.398	1.398
2	1	1.367	1.388	1.387	1.406
7	1	1.425	1.403	1.387	1.368
1	8	1.085	1.084	1.080	1.080
2	9	1.086	1.085	1.080	1.080
4	10	1.087	1.086	1.080	1.080

Convergence of Ewald summation

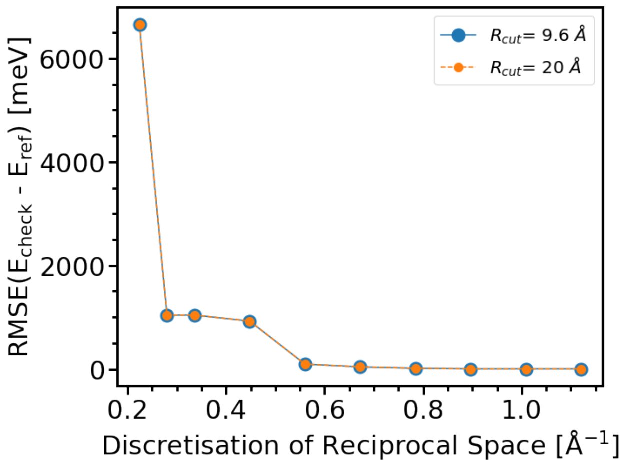

Figure S2: Convergence Ewald sum as a function of reciprocal space grid spacing. The finer the grid in reciprocal space (i.e., smaller spacing between grid points, the more accurate the result). Default Ewald settings in CP2k: η= 0.35 $Å^{-1}$. E_{ref} is taken as the energy of the last point.

Structural Stability

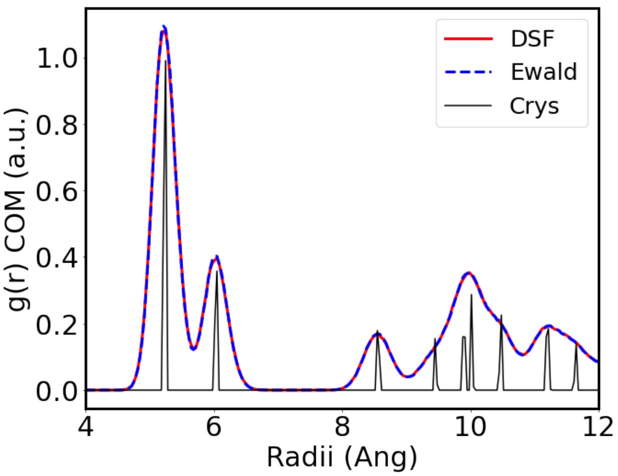

Figure S3: Structural validation of MD simulations of ANT 4x4x4 supercell. Center of mass (COM) radial distribution function (in arbitrary units) compared with experimental crystal structure for both DSF and Ewald MD trajectories

Site energies and couplings fluctuations

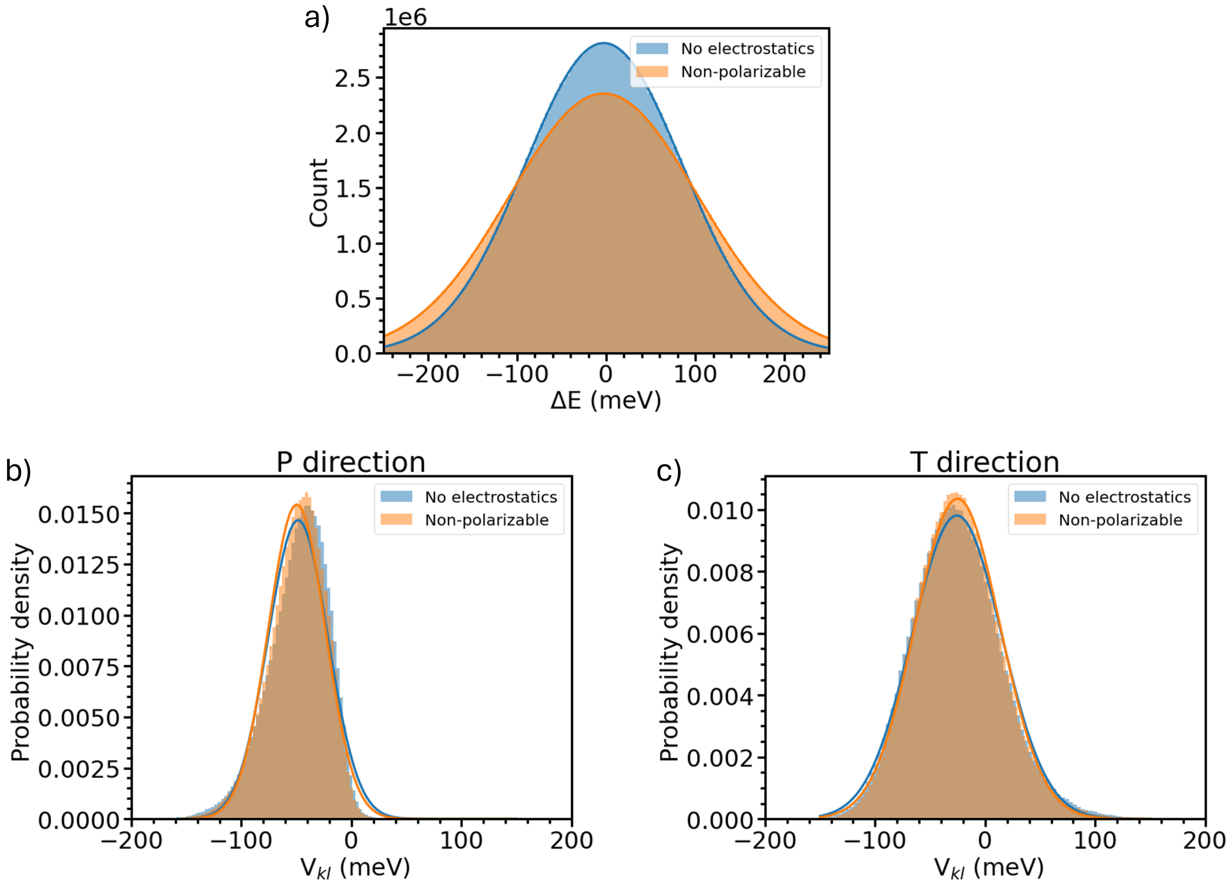

Figure S4: a) Site energy distributions with and without electrostatics and b),c) Electronic Coupling distributions in 2D simulations for molecules arranged in P and T dimers, respectively (see also Fig. S5).

Reorganization Energy

Convergence of the reorganization energy computed based on the 4-point scheme applying the polarizable force field is tested with respect to the cell size (Table S3). Structures of considered dimers are presented in Figure S5.

Table S3: Reorganization energies (λ in meV) for the CT in P and T dimers of ANT computed applying the 4-point approach computed in 4 different cell sizes.

Cell	λ^{4p}(P)	λ^{4p}(T)
2x2x2	149.9	155.9
4x4x4	173.4	175.8
6x6x6	176.8	178.6
8x8x8	176.3	177.8

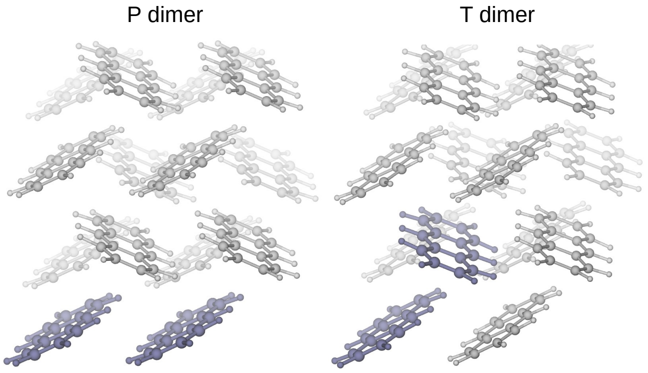

Figure S5: Structures of P and T dimers considered here.

Based on these results, the reorganization energies saturate already in computations on a 4x4x4 cell. This cell was used for reorganization energy computations from MD simulations with non-polarizable force field (with Ewald and DSF method) and with the polarizable force field.

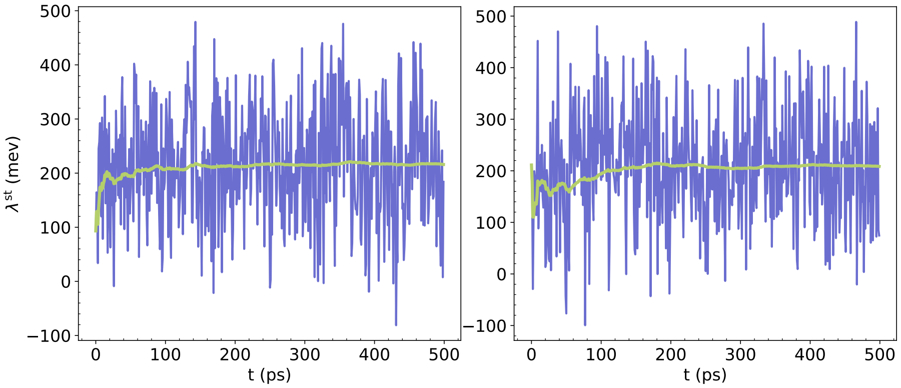

Figure S6: Instantaneous (purple) and accumulated average (green) of the vertical energy gap (Eq. 16) for reorganization energies for hole transport in P (left) and T (right) dimers of ANT computed from MD simulations with the non-polarizable force field. The average is equal to the reorganization energy λ^{st} (Eq. 15)

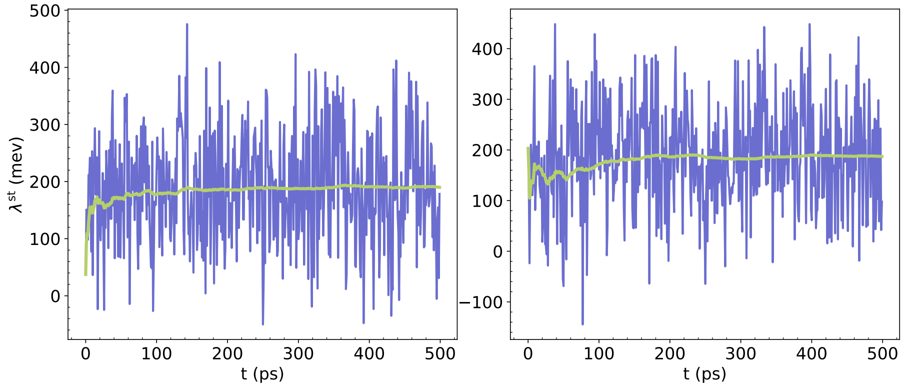

Figure S7: Instantaneous (purple) and accumulated averages (green) of the vertical energy gap (Eq. 16) for reorganization energies for hole transport in P (left) and T (right) dimers of ANT computed on an MD trajectory obtained with the non-polarizable force field applying the polarizable force field for single point computations. The average is equal to the reorganization energy λ^{st} (Eq. 15)

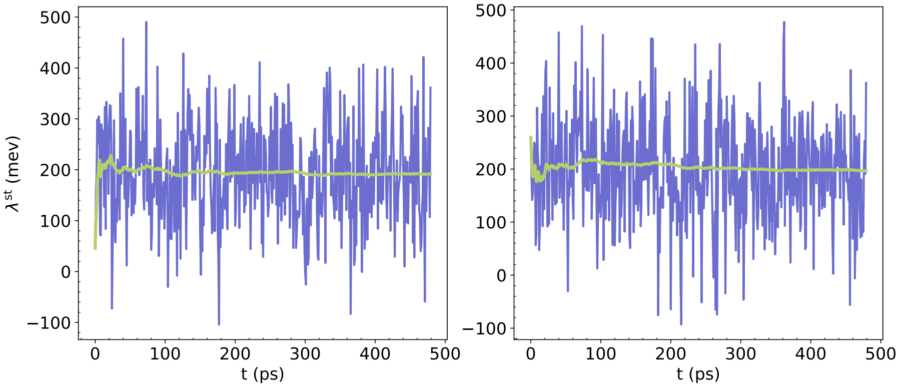

Figure S8: Instantaneous (purple) and accumulated averages (green) of the vertical energy gap (Eq. 16) for reorganization energies for hole transport in P (left) and T (right) dimers of ANT (λ^{st}) computed from MD simulations with the polarizable force field. The average is equal to the reorganization energy λ^{st} (Eq. 15)

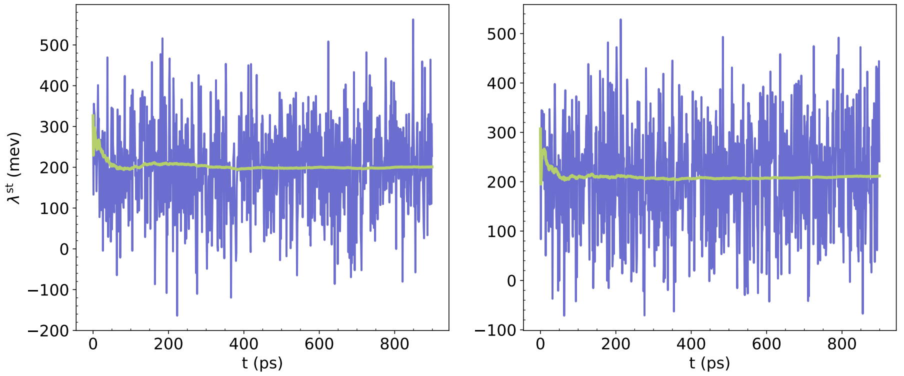

Figure S9: Instantaneous (purple) and accumulated averages (green) of the vertical energy gap (Eq. 16) for reorganization energies for hole transport in P (left) and T (right) dimers of ANT computed from MD simulations with the DSF method using the scaling factor 1.0. The average is equal to the reorganization energy λ^{st} (Eq. 15)

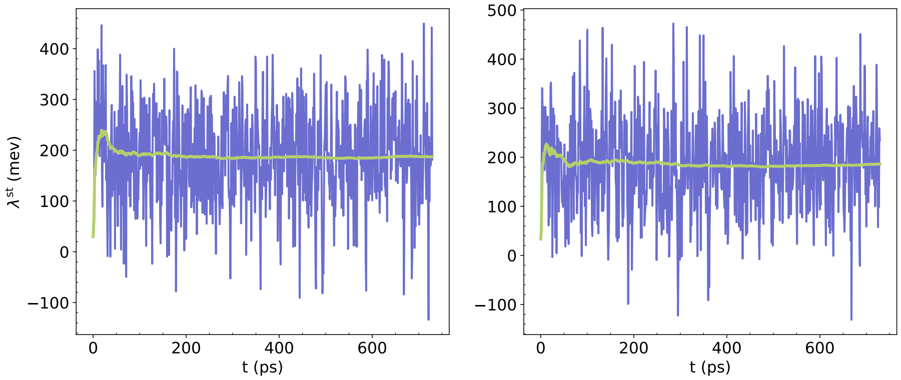

Figure S10: Instantaneous (purple) and accumulated averages (green) of the vertical energy gap (Eq. 16) for reorganization energies for hole transport in P (left) and T (right) dimers of ANT computed from MD simulations with the DSF method using the scaling factor 0.8. The average is equal to the reorganization energy λ^{st} (Eq. 15)

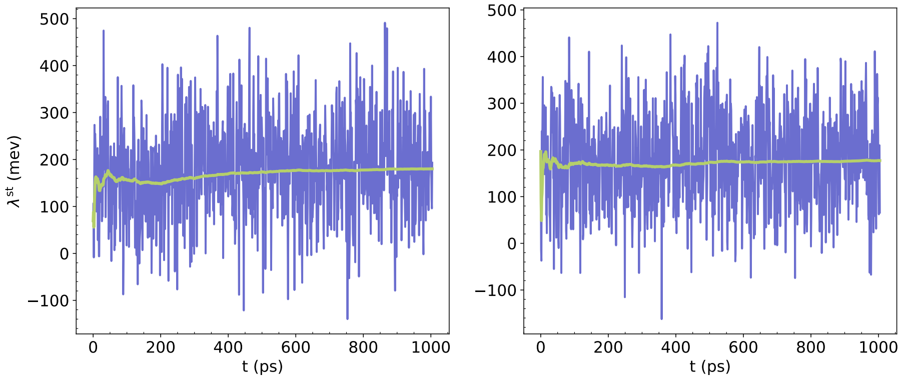

Figure S11: Instantaneous (purple) and accumulated averages (green) of the vertical energy gap (Eq. 16) for reorganization energies for hole transport in P (left) and T (right) dimers of ANT computed from MD simulations with the DSF method using the scaling factor 0.75. The average is equal to the reorganization energy λ^{st} (Eq. 15)

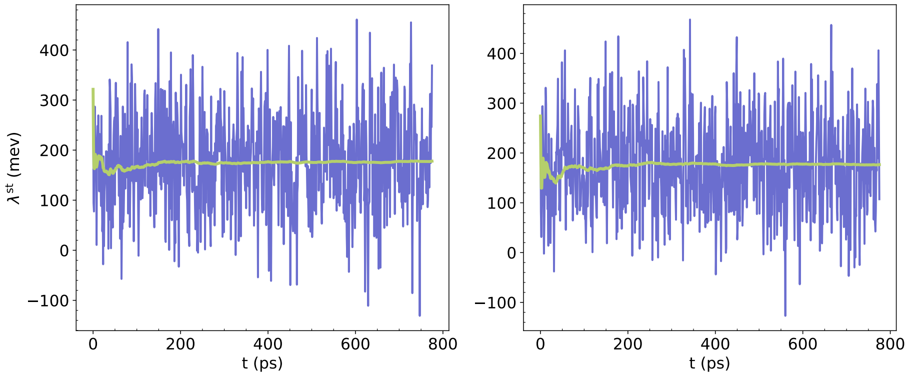

Figure S12: Instantaneous (purple) and accumulated averages (green) of the vertical energy gap (Eq. 16) for reorganization energies for hole transport in P (left) and T (right) dimers of ANT computed from MD simulations with the DSF method using the scaling factor 0.70. The average is equal to the reorganization energy λ^{st} (Eq. 15)

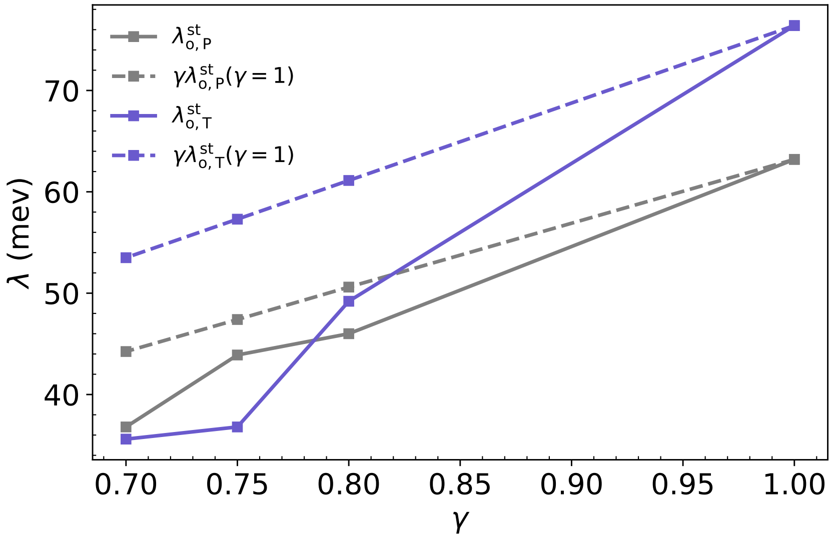

Figure S13: Outer-sphere reorganization energies for hole transport (λ^{st}_{o}, full lines) in P and T dimers computed from MD simulations with the DSF method using charge scaling factors $\gamma = 0.70, 0.75, 0.80$ and 1.00. As a guide to the eye, a linear decrease of reorganization energy with the charge scaling constant $\gamma\lambda^{st}_{o}(\gamma = 1)$ is shown in dashed lines.

Convergence tests of DSF reorganization energies

We tested the optimal cutoff values in the DSF computations of the reorganization energies in the case of 4-point scheme (λ^{4p}) at 0K, from the mean DSF energy gaps computed on an Ewald trajectory at 300K (λ^{st}), and from fluctuations of gaps (λ^{var}). For the comparison, we also provide λ^{4p} and λ^{st} obtained using Ewald summation. The results are provided in Table S4.

Table S4: Reorganization energies (λ in meV) for the hole transfer in P and T dimers of ANT computed applying the DSF method with the 4-point scheme ($\lambda^{4p}_{DSF/DSF}$), as mean energy gaps computed applying the DSF method on an Ewald trajectory ($\lambda^{st}_{DSF/Ew}$), and from the fluctuations of energy gaps ($\lambda^{var}_{DSF/Ew}$). Cutoff values in DSF computations are changed in the range from 12 to 30 . For the comparison, the values computed using Ewald method are provided as well. The errors of λ^{st} are given in parentheses. The average CPU times per single configuration energy computations are also given (t_{CPU} in s).

Dimer	R_{cut} (Å)	$\lambda^{4p}_{DSF/DSF}$	$\lambda^{st}_{DSF/Ew}$	$\lambda^{var}_{DSF/Ew}$	t_{CPU}
P	12	176.5	213.6 (6)	208.3	0.6
T		178.6	201.8 (6)	188.5	
P	14	179.8	215.6 (6)	212.3	0.9
T		181.8	203.8 (6)	191.9	
P	16	181.3	216.7 (7)	215.1	1.1
T		183	204.9 (6)	193.9	
P	18	184.1	217.4 (7)	217	1.7
T		185.7	205.6 (6)	194.8	
P	20	181.6	217.6 (7)	217.6	2
T		183.4	205.8 (6)	195.2	
P	25	181.6	217.6 (7)	217.5	3.7
T		183.6	205.9 (6)	195.6	
P	30	184.3	217.6 (7)	217.5	6.7
T		186.3	205.9 (6)	195.6	
Dimer		$\lambda^{4p}_{Ew/Ew}$	$\lambda^{st}_{Ew/Ew}$	$\lambda^{var}_{Ew/Ew}$	
P		181.3	218.6 (7)	220.3	
T		184.9	207.8 (6)	199.2	

Non-adiabatic MD additional data

Energy conservation

We tested that the dynamics with DSF included in our FOB-SH still conserves total energy as we show in Table S4. Indeed, the energy conservation, including electrostatic effects in FOB-SH is as good as without it. Note however, that the energy drift in non-adiabatic MD is higher than for simple classical MD as in Fig. 3 of the main text. For an explanation of this expected trend, see the discussion in Ref.[1]

Table S5: Energy conservation in FOB-SH

	Drift (Ha/atom/ps)	Temp. (K)
no electrostatics	4.20E-06	299.5
non-polarizable	4.00E-06	300.5
implicitly polarizable	4.20E-06	300.1

References

(1) Carof, A.; Giannini, S.; Blumberger, J. Detailed balance, internal consistency and energy conservation in fragment orbital-based surface hopping. *J. Chem. Phys.* **2017**, *147*, 214113.
